# Phylogenetic Analyses Suggest that Factors Other Than the Capsid Protein Play a Role in the Epidemic Potential of GII.2 Norovirus

**DOI:** 10.1128/mSphereDirect.00187-17

**Published:** 2017-05-17

**Authors:** Kentaro Tohma, Cara J. Lepore, Lauren A. Ford-Siltz, Gabriel I. Parra

**Affiliations:** Division of Viral Products, Food and Drug Administration, Silver Spring, Maryland, USA; Boston University School of Medicine; Chinese University of Hong Kong; University of New South Wales; Centers for Disease Control and Prevention

**Keywords:** calicivirus, noroviruses, phylogenetic analysis, transmissible gastroenteritis virus

## Abstract

Norovirus is the leading cause of acute gastroenteritis worldwide. For over two decades, a single genotype (GII.4) has been responsible for most norovirus-associated cases. However, during the winter of 2014 to 2015, the GII.4 strains were displaced by a rarely detected genotype (GII.17) in several countries of the Asian continent. Moreover, during the winter of 2016 to 2017, the GII.2 strain reemerged as predominant in different countries worldwide. This reemerging GII.2 strain is a recombinant virus that presents a GII.P16 polymerase genotype. In this study, we investigated the evolutionary dynamics of GII.2 to determine the mechanism of this sudden emergence in the human population. The phylogenetic analyses indicated strong linear evolution of the VP1-encoding sequence, albeit with minor changes in the amino acid sequence over time. Without major genetic differences among the strains, a clustering based on the polymerase genotype was observed in the tree. This association did not affect the substitution rate of the VP1. Phylogenetic analyses of the polymerase region showed that reemerging GII.P16-GII.2 strains diverged into a new cluster, with a small number of amino acid substitutions detected on the surface of the associated polymerase. Thus, besides recombination or antigenic shift, point mutations in nonstructural proteins could also lead to novel properties with epidemic potential in different norovirus genotypes.

**IMPORTANCE** Noroviruses are a major cause of gastroenteritis worldwide. Currently, there is no vaccine or specific antiviral available to treat norovirus disease. Multiple norovirus strains infect humans, but a single genotype (GII.4) has been regarded as the most important cause of viral gastroenteritis outbreaks worldwide. Its persistence and predominance have been explained by the continuous replacement of variants that present new antigenic properties on their capsid protein, thus evading the herd immunity acquired to the previous variants. Over the last three seasons, minor genotypes have displaced the GII.4 viruses as the predominant strains. One of these genotypes, GII.2, reemerged as predominant during 2016 to 2017. Here we show that factors such as minor changes in the polymerase may have driven the reemergence of GII.2 during the last season. A better understanding of norovirus diversity is important for the development of effective treatments against noroviruses.

## Abstract (precis)

Noroviruses are a major cause of gastroenteritis worldwide. Currently, there is no vaccine or specific antiviral available to treat norovirus disease. Multiple norovirus strains infect humans, but a single genotype (GII.4) has been regarded as the most important cause of viral gastroenteritis outbreaks worldwide. Its persistence and predominance have been explained by the continuous replacement of variants that present new antigenic properties on their capsid protein, thus evading the herd immunity acquired to the previous variants. Over the last three seasons, minor genotypes have displaced the GII.4 viruses as the predominant strains. One of these genotypes, GII.2, reemerged as predominant during 2016 to 2017. Here we show that factors such as minor changes in the polymerase may have driven the reemergence of GII.2 during the last season. A better understanding of norovirus diversity is important for the development of effective treatments against noroviruses.

## INTRODUCTION

Norovirus is an important cause of acute gastroenteritis worldwide, affecting people in all age groups. Norovirus infections are mainly associated with outbreaks occurring during the winter season in enclosed communities such as schools, nursing homes, or hospitals (1–4). Although norovirus infection is self-limiting in healthy individuals, it can cause severe symptoms in vulnerable populations (i.e., young children, the elderly, or immunocompromised individuals). Thus, norovirus has been associated with up to 200,000 deaths per year, mostly in children in developing countries (5, 6).

Norovirus has an ~7.5-kb single-stranded positive-sense RNA genome that is organized into three open reading frames (ORFs). The ORF1 encodes a nonstructural polyprotein that includes the RNA-dependent RNA polymerase (RdRp). The ORF2 encodes the major capsid protein (VP1), and the ORF3 encodes the minor capsid protein (VP2). VP1 is structurally divided into a shell (S) and protruding (P) domain, with the major antigenic sites located in the P domain.

Noroviruses are a highly diverse group of viruses. They are grouped into seven genogroups (GI to GVII) and more than 30 genotypes, based on the sequence differences of their VP1 proteins (7). Norovirus typing is currently based on a dual system, where sequence information from the RdRp (encoded by ORF1) and VP1 (encoded by ORF2) is required for precise molecular identification of norovirus strains (8). In several viruses, phylogenetic discrepancies have been reported in analyzing sequences from the ORF1 and ORF2 regions; thus, the junction between ORF1 and ORF2 is considered a hot spot site for norovirus recombination (9). Survey studies have shown that humans are primarily infected by GI and GII strains; however, the GII.4 genotype has been responsible for most of the norovirus-associated cases of acute gastroenteritis worldwide for almost two decades (10–12). The persistence and predominance of GII.4 strains have been linked to the constant evolution of their VP1, which results in the replacement of antigenically distinct variants every 3 to 8 years (13, 14). During the winter of 2014 to 2015, many countries in Asia reported a sudden increase in the detection of GII.17 strains, which displaced the GII.4 strains as the major cause of norovirus-associated disease (15–17). GII.17 has been rarely reported for over 30 years, but studies suggested that the fast-evolving nature of the novel GII.17 strains enabled an antigenic shift in VP1 (16, 18). The emergence of this GII.17 was also associated with the report of a new polymerase genotype, GII.P17 (15, 19). Although the acquisition of this new polymerase could have been associated with the faster evolution of the novel GII.17 strains, the mechanism of this emergence is not completely understood (18).

Just recently, Niendorf et al. reported the predominance of a recombinant GII.P16-GII.2 strain in Germany (20). The predominance of this recombinant strain was also reported in China and Japan during the 2016–2017 season, mainly in childcare centers (21–23). Although the GII.2 genotype is considered one of the four most common norovirus genotypes associated with sporadic cases in children, global analyses found that it only accounts for <2% of all strains (24). Outbreaks and sporadic infections caused by GII.2 strains have been reported since 1979 (25–29). GII.2 noroviruses have been associated with multiple different polymerases (i.e., GII.P2, GII.P12, GII.P16, GII.P21, GII.P22, GII.Pc, and GII.Ph); however, GII.P16-GII.2 noroviruses have been detected only in the last decade (30). Here, we investigated the evolutionary dynamics of GII.2 strains to elucidate the mechanisms that led to the predominance of the GII.P16-GII.2 strains during the 2016–2017 season.

## RESULTS

### Clock-like evolution of GII.2 VP1-encoding region.

**FIG 1:**
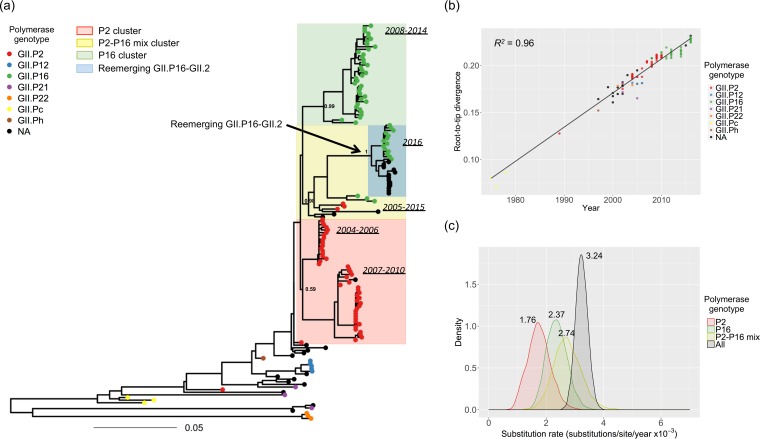
Evolutionary dynamics of the major capsid protein of GII.2 noroviruses. (a) Maximum likelihood phylogenetic tree of VP1-encoding (nt) sequences from GII.2 strains. The numbers on the ancestral nodes of the clusters represent the node support value calculated by the approximate likelihood-ratio test using PhyML. (b) Root-to-tip divergence plot of VP1 nt sequences of GII.2 strains. The *x* axis indicates isolation year, and the *y* axis shows the root-to-tip divergence on the maximum likelihood phylogenetic tree. The black line indicates a linear regression line of the root-to-tip divergence and isolation year. A total of 151 sequences were included for both analyses. Each strain is represented by a circle and colored according to its polymerase genotype. Strains whose polymerase genotype was not available are indicated in black. The genomic region used in the analyses spanned nt 5085 to 6419 relative to Snow Mountain virus (GenBank accession number AY134748). (c) The kernel density plot indicates the posterior estimates of substitution rate (substitutions/site/year) of VP1 nt sequences. To calculate the substitution rate of the VP1 associated with different polymerases, strains were clustered using the VP1 phylogenetic tree. Thus, the P2 cluster is shaded in red, the P16 cluster in green, and the P2-P16 mix in yellow. The overall rate is indicated by black shading.

To investigate the evolutionary dynamics of GII.2 VP1, sequence data were collected for GII.2 strains with a nearly complete ORF2 (1,337 nucleotides [nt]) available in the GenBank database (*n* = 151; see Table S1 in the supplemental material). The 151 VP1 sequences from the included GII.2 noroviruses were associated with seven different polymerase genotypes (GII.P2, GII.P12, GII.P16, GII.P21, GII.P22, GII.Pc, and GII.Ph), with GII.P2 (51/151, 34%) and GII.P16 (55/151, 36%) being the most commonly reported. The maximum likelihood (ML) tree showed that the GII.2 strains were mostly, but not always, grouped according to their polymerase genotypes (Fig. 1a). The reemerging GII.P16-GII.2 strains clustered together but were separated from pre-2016 GII.2 strains. Despite this apparent clustering, the pairwise p-distance of the GII.2 sequences indicated a small variation at the nt level, with no clear partition to define variants within GII.2 strains (see Fig. S1 in the supplemental material). The exceptions to this were three GII.P21-GII.2 and three GII.P22-GII.2 strains detected during 2002 to 2004 (Fig. 1). The root-to-tip divergence plot of VP1-encoding sequences showed strong clock-like evolution of the VP1, with a coefficient of determination (*R*^*2*^) value of 0.96 (Fig. 1b). The GII.P2-GII.2 strains were detected from 1989 to 2010 and were then replaced by the GII.P16-GII.2 strains that started to circulate in 2008. This replacement did not alter the evolutionary rate of VP1, which kept diverging on the linear regression line regardless of the polymerase genotypes (Fig. 1b). To determine whether the acquisition of a different polymerase affected the substitution rate, we grouped the strains based on their polymerase genotype and VP1 phylogenetic clustering. Thus, four data sets were used for these analyses: one that included all sequences, one that included strains from a P2 cluster (red shaded, Fig. 1a), one that contained strains from a P16 cluster (green shaded, Fig. 1a), and another one that contained strains with P2 or P16 but that clustered together in the phylogenetic tree (yellow shaded, Fig. 1a). Of note is that the recently reported reemerging strains, the recombinant GII.P16-GII.2 strains, were located in the P2-P16 mix cluster (blue shaded, Fig. 1a). The mean substitution rate of the overall VP1 sequences was 3.24 × 10^−3^ substitutions/site/year (95% highest posterior density [HPD] interval: 2.84 × 10^−3^ to 3.67 × 10^−3^). The mean substitution rates of strains bearing GII.P2, GII.P16, or GII.P2-P16 were similar to each other (i.e., 1.75 × 10^−3^, 2.37 × 10^−3^, and 2.74 × 10^−3^ substitutions/site/year, respectively [Fig. 1c]); however, the substitution rate of the GII.P2 strains was slightly lower than the overall rate. These substitution rates were also observed when the complete VP1 sequences were used in the analyses (Fig. S2).

10.1128/mSphereDirect.00187-17.1 FIG S1 Histogram of the pairwise p-distance of VP1 nucleotide (nt) sequences among GII.2 strains (*n* = 151). The pairwise distance data for three strains from the GII.P21 cluster (including two strains with unknown polymerase genotypes [KC998960 and AY660568] and JQ320072) and three from the GII.P22 cluster (DQ366347, AB279554, and AB279556) are indicated in black. The genomic region used spanned nt 5085 to 6713 of Snow Mountain virus (GenBank accession number AY134748). Download FIG S1, TIF file, 1 MB. Copyright © 2017 Tohma et al. 2017 Tohma et al. This content is distributed under the terms of the Creative Commons Attribution 4.0 International license.

10.1128/mSphereDirect.00187-17.2 FIG S2 Evolutionary dynamics of the major capsid protein of GII.2 noroviruses. Evolutionary dynamics of VP1 was analyzed by using full-length VP1-encoding (nt) sequences. (a) Maximum likelihood phylogenetic tree of VP1 nt sequences from GII.2 strains. The numbers on the ancestral nodes of the clusters represent the node support values calculated by the approximate likelihood-ratio test using PhyML. (b) Root-to-tip divergence plot of VP1 nt sequences of GII.2 strains. The *x* axis indicates isolation year, and the *y* axis shows the root-to-tip divergence on the maximum likelihood phylogenetic tree. The black line indicates a linear regression line of the root-to-tip divergence and isolation year. A total of 134 sequences are included for both analyses. Each strain is colored according to its polymerase genotype. Strains whose polymerase genotype was not available are indicated in black. The genomic region used in the analyses spanned nt 5085 to 6713 relative to Snow Mountain virus (GenBank accession number AY134748). (c) The kernel density plot indicates the posterior estimates of substitution rates (substitutions/site/year) of VP1 nt sequences. To calculate the substitution rate of the VP1 protein associated with different polymerases, strains were clustered using the VP1 phylogenetic tree. Thus, the P2 cluster is shaded in red, the P16 cluster in green, and the P2-P16 mix in yellow. The overall rate is indicated by black shading. Download FIG S2, TIF file, 1.9 MB. Copyright © 2017 Tohma et al. 2017 Tohma et al. This content is distributed under the terms of the Creative Commons Attribution 4.0 International license.

10.1128/mSphereDirect.00187-17.7 TABLE S1 Data set. Download TABLE S1, PDF file, 0.04 MB. Copyright © 2017 Tohma et al. 2017 Tohma et al. This content is distributed under the terms of the Creative Commons Attribution 4.0 International license.

### Small divergence of amino acid (aa) sequences during a 42-year period.

**FIG 2:**
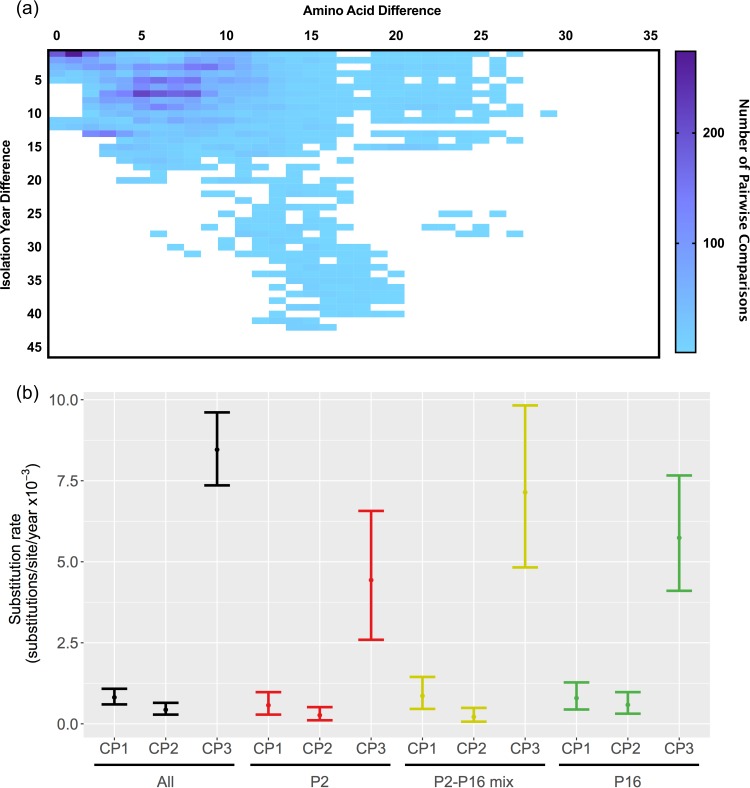
Amino acid diversification of the major capsid protein of GII.2 noroviruses. (a) Heat map indicating the accumulation of aa mutations in the partial VP1 sequences of GII.2 strains (445 amino acids) over time. The *x* axis indicates the pairwise aa mutations, and the *y* axis indicates isolation year. The number of pairwise comparisons is represented by the color gradient in the heat map. (b) The plot indicates the substitution rates (substitutions/site/year) of VP1 nucleotide sequences at each codon position. The mean and the 95% highest posterior density intervals are indicated. The substitution rates were calculated using the same sequence matrix and groups as those represented in Fig. 1.

Although nt sequences showed clock-like linear evolution, the aa substitutions did not accumulate over time (Fig. 2a). The plot of aa differences over time indicates minor divergence (≤5%, a cutoff value to define intragenotype variants) at the VP1 for almost four decades, in concordance with the static nature of non-GII.4 noroviruses (14). Only three GII.2 strains detected in Japan during 2001 to 2004 and associated with the GII.P22 polymerase showed >5% aa divergence when the complete VP1 sequence was used (Fig. S3). This discrepancy in the clock-like evolution of the nt sequences and the limited diversity at the aa level were mostly driven by the high rate of evolution presented at the third codon positions, which led to synonymous substitutions (Fig. 2b). This pattern was observed regardless of the polymerase genotypes associated with the strain (Fig. 2b). The substitution rates at the first and second codon positions were comparable among all data sets, suggesting similar rates of nonsynonymous substitution. In addition, the phylogenetic tree of VP1 inferred using aa sequences showed no clear divergence among the P2 and P2-P16 clusters, including the reemerging GII.P16-GII.2 strains (Fig. S4). Of note, the aa sequences from the reemerging GII.P16-GII.2 strains were almost identical (≤2 aa) to those from some of the GII.2 strains in the P2 and P2-P16 mix clusters detected in 2004 to 2009 (Table 1; Fig. S4). Of note is that 11 of 27 reemerging GII.2 strains, including GII.P16-GII.2/CUHK-NS-1082/2016/HKG (KY771081; full-length VP1), GII.2/CUHK-NS-1231/2016/HKG (KY421044; full-length VP1), and strains isolated in Germany (KY357450, KY357451, KY357452, KY357453, KY357454, KY357456, KY357457, KY357458, and KY357462; partial VP1), presented 100% identical capsid protein sequences compared with GII.P2-GII.2/OH06023/2006/JPN (AB662863) and GII.2/Vaals87/2005/NLD (AB281090) strains.

10.1128/mSphereDirect.00187-17.3 FIG S3 Amino acid diversification of the major capsid protein of GII.2 noroviruses. The heat map indicates the accumulation of aa mutations in the complete VP1 of GII.2 strains (543 amino acids) over time. The *x* axis indicates the pairwise aa mutations, and the *y* axis indicates isolation year. The number of pairwise comparisons is represented by the color gradient in the heat map. Download FIG S3, TIF file, 0.3 MB. Copyright © 2017 Tohma et al. 2017 Tohma et al. This content is distributed under the terms of the Creative Commons Attribution 4.0 International license.

10.1128/mSphereDirect.00187-17.4 FIG S4 Maximum likelihood phylogenetic tree of VP1 amino acid sequences of GII.2 strains. Each strain is colored according to its polymerase genotype. Strains whose polymerase genotype was not available are indicated in black. A total of 151 sequences were included in the analysis. The genomic region used spanned nt 5085 to 6419 relative to Snow Mountain virus (GenBank accession number AY134748). The numbers on the ancestral nodes of the clusters represent the node support value calculated by the approximate likelihood-ratio test using PhyML. The color shade indicates the phylogenetic clustering based on the polymerase genotype as indicated in Fig. 1. Download FIG S4, TIF file, 1.1 MB. Copyright © 2017 Tohma et al. 2017 Tohma et al. This content is distributed under the terms of the Creative Commons Attribution 4.0 International license.

**TABLE 1:** Amino acid substitution observed in the consensus VP1 sequence among phylogenetic clusters

Strain or cluster	No. of sequences	Amino acid substitution at codon:								
		71	130	303	335	344	354	364	386	400
Reemerging GII.P16-GII.2 (2016)	27	A	V	V	I (1 V)^a^	S (1G)^a^	G	A	N	E
P2-P16 mix cluster^b^ (2005–2015) (without reemerging strains)	9	A	V	V	I (2 V)^a^	S	G	A	N	E
P16 cluster^b^ (2008–2014)	38	S	V	V	V	T (1 I, 2 A)^a^	A	A	N	D (2 E)^a^
P2 cluster^b^ (2007–2010)	29	A	I (4 V)^a^	I (1 V)^a^	I	S	G	A	S	E
P2 cluster^b^ (2004–2006)	18	A	V	V	V	S	G	S (1P)^a^	N	E

a Data in parentheses represent minor variants within the cluster.

b Phylogenetic clustering as shown in Fig. 1, assigned based on the phylogenetic clustering of the VP1-encoding sequence and the associated polymerase genotype.

### Diversifying selection through the evolution of GII.2 VP1.

**FIG 3:**
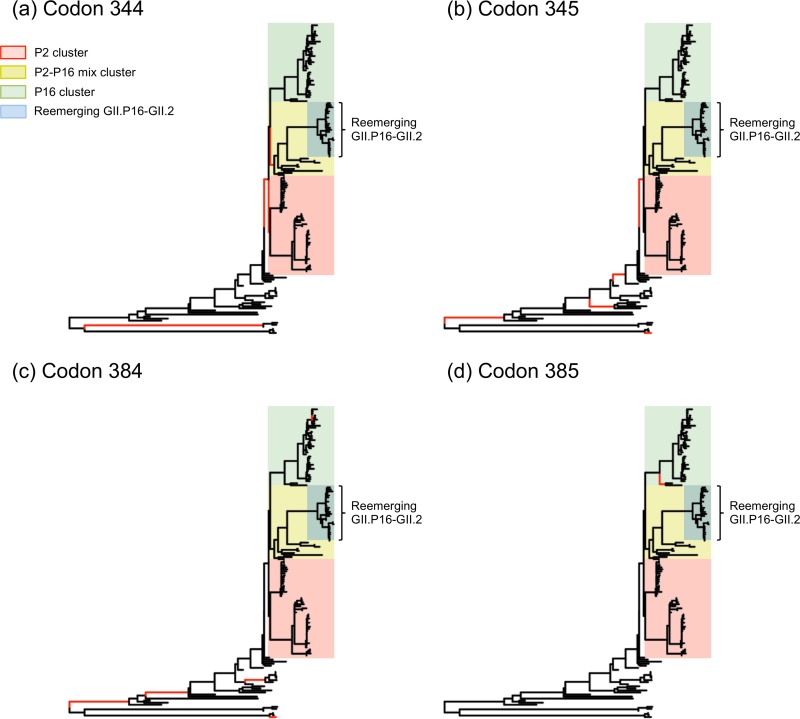
Positive evolution of the major capsid protein of GII.2 noroviruses. Maximum likelihood trees of VP1 nucleotide (nt) sequences of GII.2 indicate the branches under possible positive selection. The branches with evidence of positive selection (empirical Bayes factor of >10, *P* < 0.05; estimated using the mixed-effect model of evolution [MEME] method) at codon positions 344 (a), 345 (b), 384 (c), and 385 (d) are represented by red branch lines. The color shade indicates the phylogenetic clustering based on the polymerase genotype as indicated in Fig. 1. Reemerging GII.P16-GII.2 strains are indicated in blue.

To determine whether positive selection on the VP1 led to the reemergence and posterior predominance of the GII.P16-GII.2 strains, we performed selection analyses using internal fixed-effect likelihood (iFEL) and mixed-effect model of evolution (MEME) methods (31, 32). The iFEL test estimates the site-by-site positive selection along the internal branches of the phylogenetic tree, assuming the same nonsynonymous/synonymous substitution ratio on the branches. The MEME test, on the other hand, estimates the branch-to-branch positive selections at individual sites to identify the episodic selections (episodes of diversifying selection in a portion of branches). As a result, significant positive selection (*P* < 0.05) was detected at eight codon sites, among which codon site 345 was detected by both methods (Table 2). Five of those eight sites were located on the P2 domain of the virus particle. All of the codons identified using the partial VP1 sequences were also detected as positively selected using the full-length VP1 data set, which showed three additional positively selected sites on the P2 domain (Table S2). Of note, only a few positive sites were observed on the branches diversifying the clusters, and empirical Bayes factor (EBF) data indicated no evidence of positive selection on the branches of reemerging GII.P16-GII.2 strains (Fig. 3).

10.1128/mSphereDirect.00187-17.8 TABLE S2 Codon position of complete VP1 sequences (1,629 nt) with positive selection. Download TABLE S2, PDF file, 0.03 MB. Copyright © 2017 Tohma et al. 2017 Tohma et al. This content is distributed under the terms of the Creative Commons Attribution 4.0 International license.

**TABLE 2:** Codon position of VP1 sequences (1,337 nucleotides) with positive selection

Positively selected site	Method(s) (*P* value)	Domain
24	MEME^a^ (0.015)	N terminus
78	iFEL^b^ (0.015)	Shell
275	MEME (0.016)	P1
344	MEME (0.039)	P2 (surface)
345	iFEL (0.01), MEME (0.020)	P2 (surface)
384	MEME (0.01)	P2 (surface)
385	MEME (0.024)	P2 (surface)
397	iFEL (0.045)	P2 (surface)

a MEME, mixed-effect model of evolution.

b iFEL, internal fixed-effect likelihood methods.

Taking the data together, the reemergence of GII.P16-GII.2 strains during 2016 did not involve changes in the substitution rate or acquisition of aa mutations in the major capsid protein.

### Phylogeny of RdRp-encoding region.

**FIG 4:**
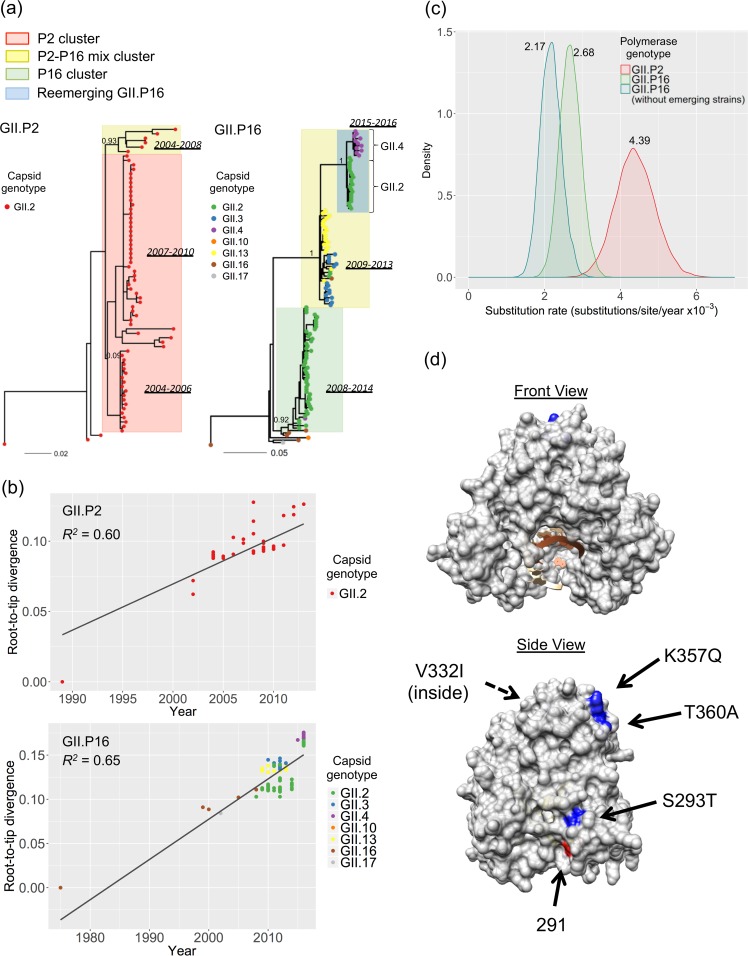
Evolutionary dynamics of the GII.2 polymerase. (a) Maximum likelihood trees of RdRp-encoding (nt) sequences of strains GII.P2 (*n* = 72) and GII.P16 (*n* = 131). Phylogenetic clusters based on VP1 nt sequences (Fig. 1) are shown here; the P2 cluster is represented in red, the P16 cluster in green, and the P2-P16 mix cluster in yellow. Reemerging GII.P16 strains, including GII.P16-GII.2 and GII.P16-GII.4, are indicated in blue. Each strain is represented by a circle, with different colors according to the associated capsid genotypes; GII.P2-GII.2 and GII.P16-GII.2 strains are indicated by red and green, respectively. The numbers on the ancestral nodes of the clusters represent the node support value calculated by the approximate likelihood-ratio test using PhyML. (b) The root-to-tip divergence plot of RdRp-encoding (nt) sequences of GII.P2 and GII.P16 strains. The *x* axis indicates isolation year, and the *y* axis shows the root-to-tip divergence on the maximum likelihood phylogenetic tree. The black lines indicate a linear regression line of the root-to-tip divergence and isolation year. Each strain is colored according to its capsid genotype. The genomic regions used spanned nt 4385 to 5104 relative to Snow Mountain virus (GenBank accession number AY134748). (c) The kernel density plot indicates the posterior estimates of substitution rate (substitutions/site/year) among GII.P2, GII.P16, and GII.P16 without reemerging strains. (d) Structural mapping of the amino acid (aa) substitutions in the reemerging GII.P16-GII.2 strains. Conservative mutations in the RdRp aa sequence from the reemerging GII.P16 strains compared to pre-2016 GII.P16 strains (P16 cluster and P2-P16 mix cluster) are mapped on the structural model of a GII.P4 RdRp (PDB number 4QPX). The molecular model was visualized using Chimera v.1.11. aa substitutions are indicated in blue. Residue 291, a residue that was shown by Bull et al. (46) to alter the incorporation rate of GII.4 polymerases, is indicated in red.

We also performed phylogenetic analyses of the RdRp region of GII.P2 and GII.P16, the most commonly detected polymerase genotypes of GII.2. A total of 72 GII.P2 sequences and 131 GII.P16 sequences were collected for the analyses (Table S1). GII.P2 was associated with the GII.2 capsid, and GII.P16 was associated with seven different capsid genotypes (GII.P16-GII.2, GII.P16-GII.3, GII.P16-GII.4, GII.P16-GII.10, GII.P16-GII.13, GII.P16-GII.16, and GII.P16-GII.17). The phylogenetic clustering shown on the VP1 nt tree was also observed in the tree of RdRp nt sequences of GII.P2 and GII.P16, although the support for the P2 cluster in the tree of GII.P2 was weak (Fig. 4a). The root-to-tip divergence plot showed moderate clock-like evolution over time (*R*^*2*^ = 0.60 and *R*^*2*^ = 0.65 for GII.P2 and GII.P16, respectively) (Fig. 4b). The reemerging GII.P16-GII.2 strains were clustered together with the emerging recombinant GII.P16-GII.4_Sydney strains detected in 2015 to 2016 (Fig. 4a and b). It is difficult to determine whether these two strains came from the same parental strain or whether one originated from the other. However, the phylogenetically similar GII.P16-GII.2 strains have been circulating since 2011, and the regression line in the root-to-tip analyses of both RdRp and VP1 shows a linear evolution from 2011 to the 2016–2017 GII.P16-GII.2 strains. This suggests that the GII.4_Sydney strain acquired GII.P16 from GII.P16-GII.2 strains. The substitution rate of all GII.P16 strains was 2.68 × 10^−3^ substitutions/site/year (95% HPD interval: 2.12 × 10^−3^ to 3.21 × 10^−3^). It slightly decreased when reemerging GII.P16 strains detected during 2015 to 2016 (GII.P16-GII.2 and GII.P16-GII.4_Sydney) were removed from the analyses (2.17E−3 substitutions/site/year [95% HPD interval: 1.64 × 10^−3^ to 2.72 × 10^−3^]), but their 95% HPD intervals overlapped with each other, suggesting no significant difference (Fig. 4c). The substitution rate of the RdRp-encoding sequence of GII.P2 (4.39E−3 substitutions/site/year [95% HPD interval: 3.41 × 10^−3^ to 5.44 × 10^−3^]) was higher than that of GII.P16 (Fig. 4c), but the aa tree showed no variation in GII.P2 (Fig. S5a). On the other hand, the aa tree of GII.P16 showed phylogenetic clustering similar to what was observed in the nt tree. The P2-P16 mix cluster and the P16 cluster were clearly separated in the aa tree of GII.P16 (Fig. S5b). The reemerging GII.P16 strains circulating in 2015 to 2016 clustered separately from the ones detected pre-2013, and their RdRp consensus sequences presented 4 aa substitutions compared with the other GII.P16 strains detected pre-2013 (Table S3). Three of the aa substitutions were located on the surface of the RdRp structure (Fig. 4d). This suggests that modification in the RdRp may play some role in the predominance of GII.P16-GII.2 strains.

10.1128/mSphereDirect.00187-17.5 FIG S5 Maximum likelihood trees of the RdRp amino acid sequences. Trees were built using GII.P2 (*n* = 72) (a) and GII.P16 (*n* = 131) (b) strains. Phylogenetic clusters based on VP1 nt sequences (Fig. 1) are shown here; the P2 cluster is represented in red, the P16 cluster in green, and the P2-P16 mix cluster in yellow. Reemerging GII.P16 strains, including GII.P16-GII.2 and GII.P16-GII.4, are indicated in blue. Each strain is represented by a circle, with different colors according to the associated capsid genotypes; GII.P2-GII.2 and GII.P16-GII.2 strains are indicated by red and green, respectively. The numbers on the ancestral nodes of the clusters represent the node support value calculated by the approximate likelihood-ratio test using PhyML. Download FIG S5, TIF file, 1.5 MB. Copyright © 2017 Tohma et al. 2017 Tohma et al. This content is distributed under the terms of the Creative Commons Attribution 4.0 International license.

10.1128/mSphereDirect.00187-17.9 TABLE S3 Amino acid substitution observed in the RdRp sequence of reemerging GII.P16 strains. Download TABLE S3, PDF file, 0.03 MB. Copyright © 2017 Tohma et al. 2017 Tohma et al. This content is distributed under the terms of the Creative Commons Attribution 4.0 International license.

### Divergence history of VP1 and RdRp.

The time of the most recent common ancestor (tMRCA) was determined and summarized in the time-measured maximum clade credibility (MCC) trees for both VP1-encoding and RdRp-encoding sequences (Fig. S6). The times of divergence of the phylogenetic clusters among VP1 and RdRp MCC trees were comparable, indicating the coevolution of both regions. The tMRCA of the recently reemerged GII.P16-GII.2 strains, based on partial VP1 and partial RdRp sequences of GII.P16, dated back to 2012 to 2013. Both MCC trees clearly showed that these strains evolved from the GII.P16-GII.2 strains reported in 2011 to 2012.

10.1128/mSphereDirect.00187-17.6 FIG S6 Maximum clade credibility (MCC) tree of GII.2 strains. VP1-encoding sequences of GII.2 strains (*n* = 151) (a), RdRp-encoding sequences from GII.P2 strains (*n* = 72) (b), and GII.P16 strains (*n* = 131) (c) were used for tree reconstructions. Each strain in the VP1 MCC tree is colored according to its polymerase genotype (a), and each strain in the RdRp MCC trees is colored by its capsid genotype (b and c). Strains whose polymerase genotype was not available are indicated in black. Reemerging GII.P16-GII.2 strains are indicated by blue shading. Arrows indicate the means and 95% highest posterior density intervals of the time of the most recent common ancestor. Phylogenetic clustering is indicated by color as described for Fig. 1. The numbers on the ancestral nodes of the clusters represent the posterior probability values of the nodes. The *x* axis and branch length represent the year. Estimated divergence time is indicated on the node of common ancestors of phylogenetic clustering. Download FIG S6, TIF file, 2.3 MB. Copyright © 2017 Tohma et al. 2017 Tohma et al. This content is distributed under the terms of the Creative Commons Attribution 4.0 International license.

## DISCUSSION

For over two decades, GII.4 noroviruses have been the predominant genotype circulating in humans. This dominance in human population has been attributed to the continuous replacement of antigenically distinct intragenotype variants, which can escape from herd immunity (13, 14). In contrast, none of the other genotypes have been shown to possess this advantage, as their VP1 aa sequences remain almost invariable (or static) after decades of circulation in the human population (14). During the 2014–2015 season, the GII.4 genotype was unexpectedly replaced by the GII.17 genotype in some Asian countries (15, 17, 33–35). The predominance of GII.17 strains did not last long, as during the winter of 2016 to 2017, the GII.2 genotype was shown to predominate in Germany, China, Japan, Taiwan, and Hong Kong (20–23, 36–38).

During the last 2 years, extensive research has been done to understand the driving forces that led GII.17 to become predominant in various Asian countries (16, 18). The major hypothesis is that acquisition of a novel polymerase could have driven rapid evolution of the VP1 protein that resulted in modifications of the capsid protein that increased its infectivity potential. The support for this hypothesis is based on the following: (i) the GII.P17 polymerase was described for the first time with the novel strains (15, 19); (ii) the VP1 protein from the novel GII.17 presents multiple differences (>10%), including aa insertions and deletions, compared with previous GII.17 viruses (15, 19); (iii) the people infected with GII.P17-GII.17 viruses were significantly older than the ones infected with GII.4 viruses (34); (iv) the novel GII.17 presented a broad pattern of binding to histoblood group antigens (susceptibility factors for human noroviruses) (39); and (v) despite being detected worldwide, the novel GII.17 strain predominated only in Asian countries, suggesting race-related susceptibility. Of note, despite all these observations, the GII.17 strains present overall a very low number of aa changes in their VP1 (14), and these “novel GII.17 strains” have been found to have been circulating as far back as the 1970s (40).

Although a large number of complete VP1 sequences are available in the public databases for GII.17, most of them (136/143) correspond to the novel GII.P17-GII.17 strain (14) and extensive analyses of the differences in the rate of evolution among the different variants of GII.17 were not possible (18). Since a better (time-ordered) sequence database is available for GII.2 in GenBank, we investigated the evolutionary history of this genotype to better understand the recent predominance of the GII.P16-GII.2 strains. Our analyses show that the VP1 nt sequences of GII.2 viruses have been evolving linearly for decades, regardless of the polymerase genotypes associated with that VP1, and with similar substitution rates among phylogenetic clusters. GII.P2-GII.2 strains have been shown to be circulating at high frequencies in different areas of Japan since 2004 (27), and GII.P16-GII.2 strains have been detected since 2008 (30). Comparisons of the substitution rates in each of the different strains have shown very little variation in the VP1 evolution of the GII.2 strains.

The VP1 protein from GII.2 strains has been stable at the aa level for more than 40 years. Although few aa substitutions were noted in the VP1 sequences, and some of them mapped in locations that would resemble the antigenic sites described for GII.4 viruses, the VP1 aa sequences of the reemerging GII.P16-GII.2 strain were almost (>99.5%) identical with those of previously detected GII.P2-GII.2 strains. In addition, we found no evidence that episodic diversifications (estimated throughout the internal branches of the VP1 nt tree) acted positively on the reemergence of the GII.P16-GII.2 viruses. Using virus-like particles (VLPs) from time-ordered GII.2 variants collected from 1975 to 2010, Swanstrom et al. have shown that the cross-blockade titers remained the same (41). This suggests that the aa differences found among different GII.2 strains have minimal impact on the antigenicity of GII.2 viruses. Taken together, these data suggest that neither immune pressure nor the presence of different polymerase genotypes altered the evolutionary pattern of the VP1.

Using large-scale genomics, it has been recently shown that non-GII.4 noroviruses present minor variations in their VP1 over decades (static genotypes), while GII.4 noroviruses evolve by continuous replacement of antigenically distinct variants, thus infecting a larger population (14). A concordance between the divergence times seen using VP1 or RdRp data indicates that the GII.P16-GII.2 strain, which is predominating during the 2016–2017 season, is not a novel recombinant and that this strain had evolved following a linear root-to-tip regression line from the GII.P16-GII.2 strains circulating back in 2011 to 2012. Thus, if the GII.P16-GII.2 strains were circulating years before their predominance, and if no differences were found in the evolutionary patterns of VP1 and its antigenicity, then what are the forces behind their sudden predominance over the evolving GII.4 genotype in certain countries?

Our analyses of the RdRp region suggest that GII.P2 strains evolve at a higher substitution rate at the RdRp-encoding region than any GII.P16 strains; however, sequence and epidemiological data suggest that GII.P16-GII.2 strains replaced GII.P2-GII.2 strains. This seems contradictory; however, the predominance of GII.P16-GII.2 viruses in certain countries (20, 21) coincided with the detection of a novel GII.4 strain, GII.P16-GII.4_Sydney (20, 42–44), suggesting that this GII.P16 polymerase could have a positive impact on the fitness of the virus. Indeed, using the murine norovirus model, Arias et al. have shown that the fidelity of the polymerase could influence the transmissibility of the virus (45). Analysis of the partial RdRp sequences indicated that GII.P2 strains are very stable in their aa sequence; however, reemerging GII.P16 strains presented 4 conservative aa substitutions compared with the pre-2016–2017 strains. These mutations in the RdRp region were located on the surface of the polymerase and could have altered the polymerase kinetics or fidelity of the reemerging GII.P16 strains. Although this is a very low number of aa differences, Bull et al. have shown that single-point mutations can affect the biological properties of the RdRp from different GII.4 strains (46). Interestingly, one of the four conservative aa substitutions, S293T, is very close to residue 291, which was shown by Bull et al. (46) to alter the kinetic activity of GII.4 polymerases. Thus, further studies on the phenotype of the different mutations within the RdRp are needed for better understanding of polymerase kinetics and/or fidelity and of their link to transmissibility and pandemic impact.

A phylogenetic clustering of norovirus genotypes (immunotypes) has been recently used to explain the pattern of norovirus reinfection recorded in children and adults (14). Of note, GII.2, GII.4, and GII.17 all classify into different immunotypes. Thus, an additional possible explanation for the reemergence of GII.P16-GII.2 is that GII.4 might have exhausted all possible VP1 variants and that the herd immunity incidentally selected genotypes from other immunotypes to prevail in the human community. Most of the GII.P16-GII.2 outbreaks have been reported to occur in childcare facilities or elementary schools in Germany (39 of 69 norovirus-associated outbreaks), Japan (30 of 74 reported outbreaks), and Taiwan (67% of 108 cluster cases) (20, 21, 36). Thus, “GII.2-naïve” populations mostly seem to be affected by the reemerging GII.P16-GII.2 strains without changes in the VP1. As the non-GII.4 are static genotypes and are unable to evolve antigenically, a non-GII.4 genotype would prevail for only a short period, possibly in limited areas and populations, before shifting to another genotype. This pattern is largely represented by the norovirus epidemiological landscape seen in the last 2 to 3 seasons.

Our results need careful interpretation because bioinformatics analysis reflects the use of different models to explain the evolutionary process, which could not be evidenced by real phenotypic differences. *In vitro* assays such as neutralization assays and replication assays are required to determine the phenotypic differences in both the antigenicity and polymerase kinetics of the reemerging GII.P16-GII.2 viruses. In addition, only a partial sequence of RdRp region was analyzed whereas other nonstructural proteins and VP2 may also play a role in viral replication and capsid assembly of norovirus (47, 48). Comprehensive analysis using full-length sequences may shed light on the evolutionary dynamics of the emerging/reemerging norovirus.

Our analyses indicated that the evolution of GII.2 VP1 was not influenced by acquisition of a different RdRp. The GII.P16-GII.2 strains have been circulating in the human population since (at least) 2008, and the current GII.P16-GII.2 strains did not show any differences in VP1 aa sequences (and thus antigenicity) that could explain their sudden predominance. Only a slight difference was found in RdRp aa sequences between reemerging and pre-2016 GII.P16 strains that could have led to their predominance. Continuing surveillance alongside complete genome information could help in the understanding of norovirus evolutionary mechanisms and of genotype replacement and, ultimately, in the control and development of effective vaccines against norovirus disease.

## MATERIALS AND METHODS

### Dataset.

Sequence data of GII.2 strains with a nearly complete ORF2 (nucleotide [nt] position 5085 to 6419 relative to Snow Mountain virus [GenBank accession number AY134748]) region that were available in the GenBank database were retrieved and aligned (*n* = 151 as of 8 February 2017; see Table S1 in the supplemental material). The data set included strains detected from 1976 to 2016 and the reemerging GII.P16-GII.2 strains detected during the 2016-2017 season (20, 37). The capsid and polymerase genotypes (when available) were confirmed using the online-based norovirus Typing Tool (8). In addition, we generated another data set (*n* = 134) using complete ORF2 sequences that did not include the reemerging GII.P16-GII.2 strains (nt positions 5085 to 6713; Table S1). All sequences were subjected to multiple alignment using ClustalW as implemented in MEGA7 (49).

### Phylogenetic analysis.

A ML phylogenetic tree of ORF2 sequences was reconstructed using the PhyML online tool (http://www.atgc-montpellier.fr/phyml/). The best substitution model (general time-reversible model with rate variation among sites and a proportion of invariable sites [GTR+G+I]) was selected according to the corrected Akaike information criterion (AICc) implemented in MEGA7. Node support was evaluated by the approximate likelihood-ratio test (50). The reconstructed ML tree was visualized using R v3.3.2 and the ape package (51, 52).

The aa sequences were translated from nt sequences using MEGA7. The ML trees of aa sequences were also estimated by using THE PhyML online tool.

### Time-measured phylogenetic analysis.

To observe the clock-like nature of the evolution of VP1 (encoded by ORF2), the root-to-tip divergence was calculated using the inferred ML tree and isolation years of each strain using TempEst v1.5 (53). The best-fitting root option was used to obtain the best correlation of the root-to-tip divergence. Calculated root-to-tip divergence was plotted using R v3.3.2 and the ggplot2 package. To reconstruct the evolutionary history of GII.2 VP1-encoding sequences, time-measured phylogenetic analysis was performed using the Bayesian Markov Chain Monte Carlo (MCMC) framework as implemented in BEAST v1.8.3 (54). The clock models (strict or relaxed lognormal clock) and tree priors (constant population size, exponential growth, or skyline) were tested, and the best models were selected on the basis of the model selection procedure using path-sampling/stepping stone-sampling marginal-likelihood estimation (55). The models used are summarized in Table S4. The MCMC runs were performed until all the parameters reached convergence (effective sample size, >200). MCMC runs were analyzed using Tracer v1.6 (http://tree.bio.ed.ac.uk/software/tracer/). The initial 10% of the logs from the MCMC run were removed before summarizing the posterior values. The density of the posterior values of the substitution rates was plotted using R and ggplot2. The MCC tree was reconstructed using the posterior set of trees and TreeAnnotator v1.8.3 and was visualized using R v3.3.2.

10.1128/mSphereDirect.00187-17.10 TABLE S4 Evolutionary models used in BEAST analyses. Download TABLE S4, PDF file, 0.03 MB. Copyright © 2017 Tohma et al. 2017 Tohma et al. This content is distributed under the terms of the Creative Commons Attribution 4.0 International license.

### Accumulation of aa substitutions over time.

To visualize the accumulation of aa substitutions within GII.2 VP1 over time, the pairwise aa differences and the timespan of isolation were calculated as indicated elsewhere (14). Heat map plots were calculated for all GII.2 VP1 sequences using GraphPad Prism version 7 (GraphPad Software, Inc., La Jolla, CA, USA), with the values from each cell representing the number of strains compared. The aa substitutions in the P domain were mapped on the structural model of the GII.2 Snow Mountain virus (Protein Data Bank [PDB] number 4RPB [56]) using UCSF Chimera v 1.11 (57).

### Selection analysis.

Diversifying selection of the VP1-encoding sequence through its evolutionary history was analyzed by using the iFEL model and MEME methods (31, 32). We aimed to detect codon sites under positive selection (i.e., more nonsynonymous substitutions than synonymous substitutions) during the evolution. We focused on the internal branches under positive selection, especially the branches diversifying into the clusters by the polymerase genotypes, to assess the influence of recombination on the adaptive evolution of VP1. Significant positive selection was indicated by *P* values of <0.05 in both methods. The evidence of positive selection on the branches was indicated by EBF values of >10 in MEME.

### Phylogenetic analysis of RdRp region.

Partial RdRp sequences of GII.P2 and GII.P16 (nt positions 4385 to 5104, relative to Snow Mountain virus) were retrieved from GenBank, with other capsid genotypes added to make a total of 72 GII.P2-GII.2 sequences (detected from 1994 to 2013) and 131 GII.P16 sequences (including GII.P16-GII.2, GII.P16-GII.3, GII.P16-GII.4, GII.P16-GII.10, GII.P16-GII.13, GII.P16-GII.16, and GII.P16-GII.17 and those detected from 1975 to 2016; Table S1). Data corresponding to the ML phylogenetic tree, root-to-tip divergence, and time-measured phylogenetic tree of each polymerase genotype were estimated as indicated above for the VP1-encoding sequences.

The aa substitutions on the RdRp of reemerging GII.P16 strains were mapped on the structural model of the GII.4 virus (PDB number 4QPX [58]) using UCSF Chimera v 1.11 (57).
