# Supplementary figures and images for: Phylogenetic Analyses Suggest that Factors Other Than the Capsid Protein Play a Role in the Epidemic Potential of GII.2 Norovirus
Source: mSphere. 2017 May 17;2(3):e00187-17. doi: 10.1128/mSphereDirect.00187-17 (PMC5437133; doi:10.1128/mSphereDirect.00187-17)

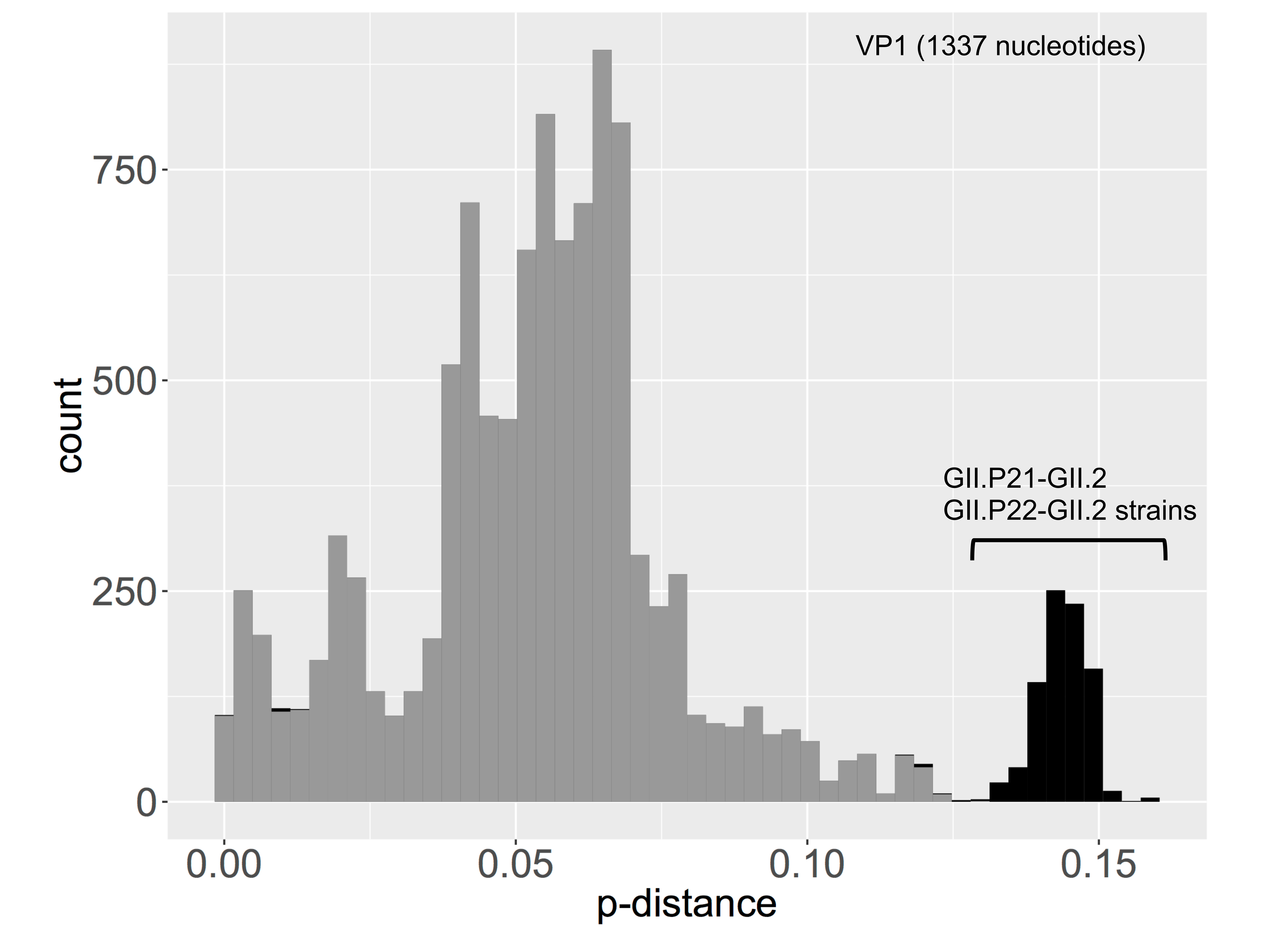

Supplement: FIG S1 [file sph003172286sf1.tif]

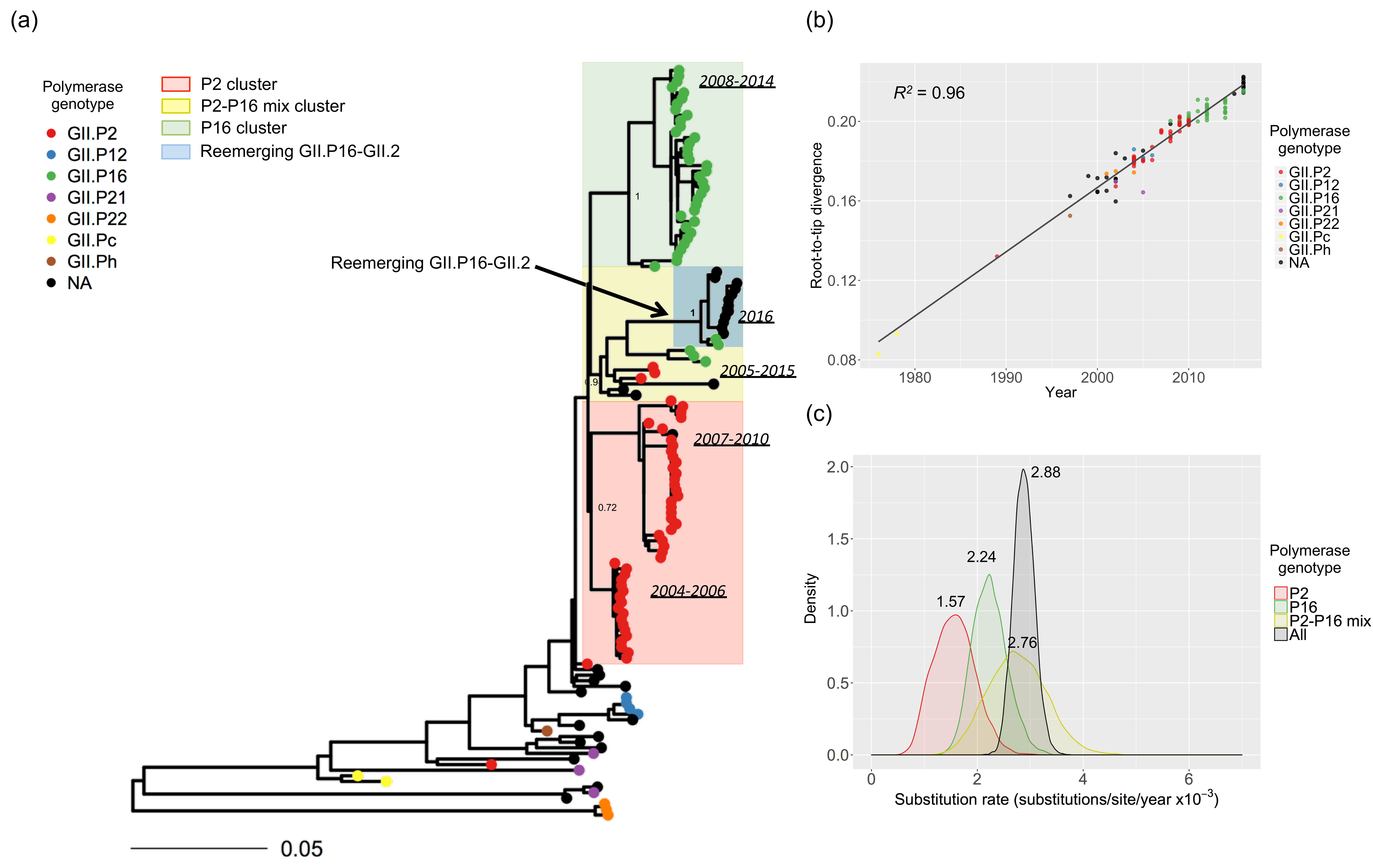

Supplement: FIG S2 [file sph003172286sf2.tif]

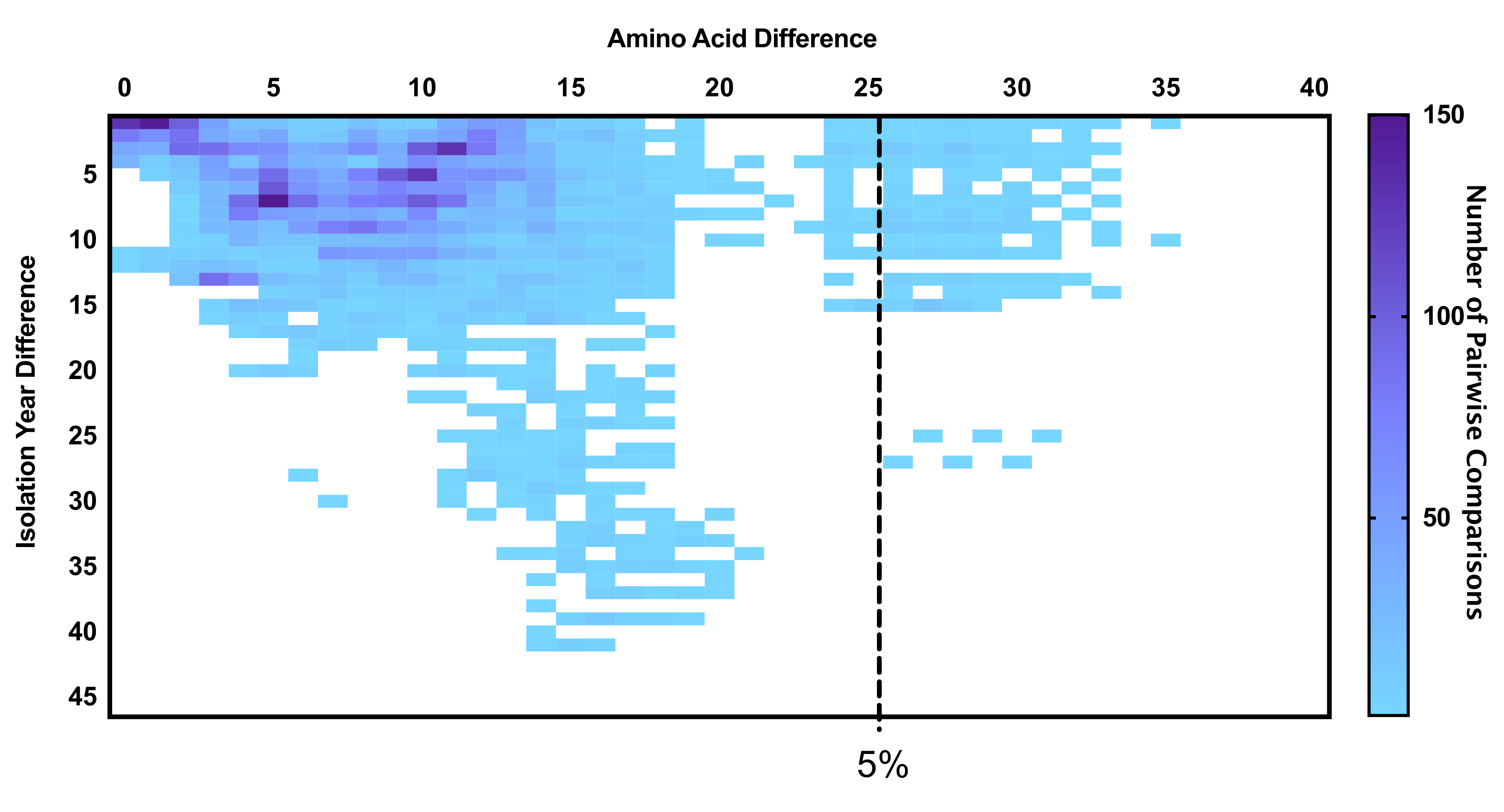

Supplement: FIG S3 [file sph003172286sf3.tif]

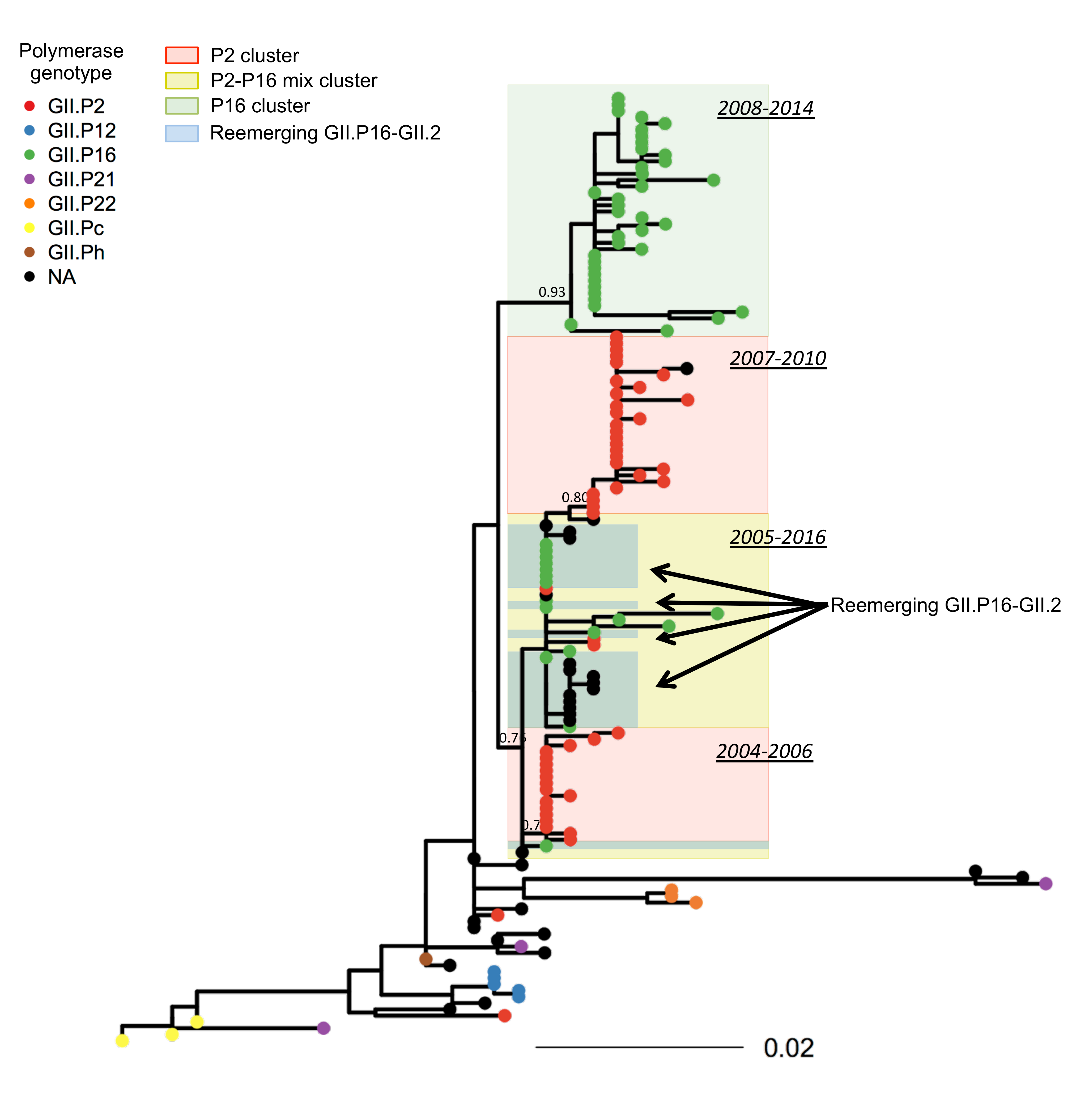

Supplement: FIG S4 [file sph003172286sf4.tif]

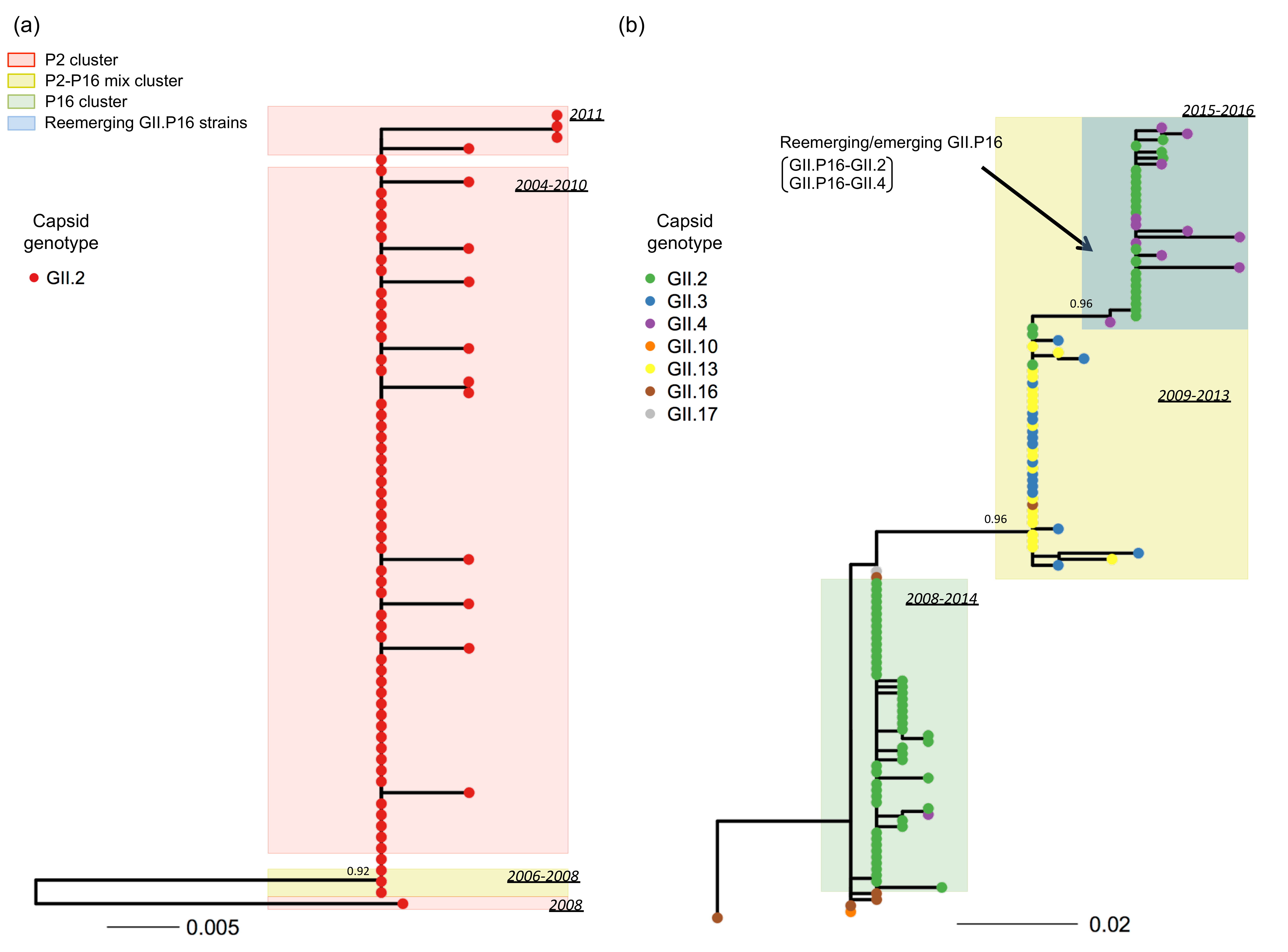

Supplement: FIG S5 [file sph003172286sf5.tif]

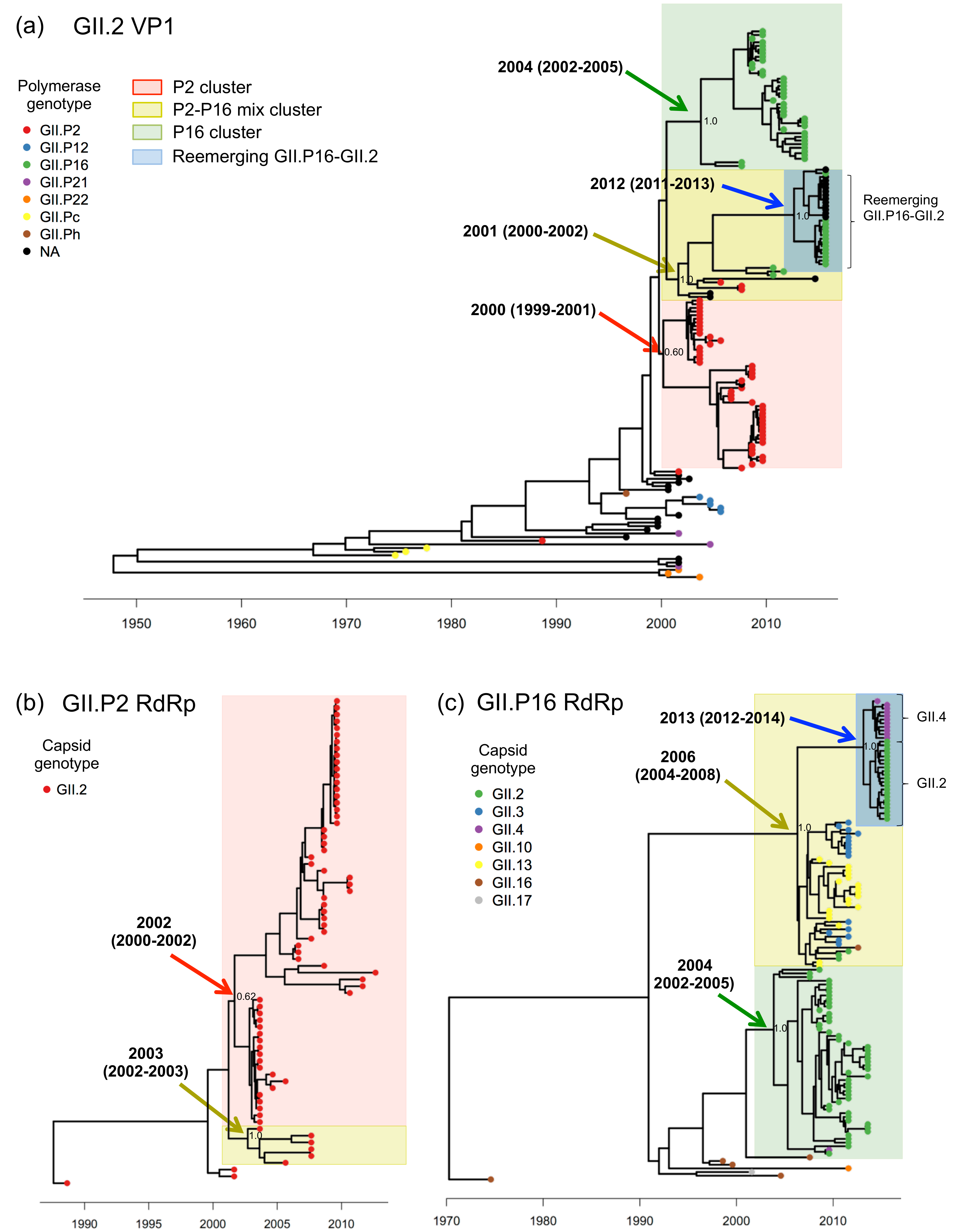

Supplement: FIG S6 [file sph003172286sf6.tif]
