# Supplementary material for: Phylogenetic Analyses Suggest that Factors Other Than the Capsid Protein Play a Role in the Epidemic Potential of GII.2 Norovirus
Source: mSphere. 2017 May 17;2(3):e00187-17. doi: 10.1128/mSphereDirect.00187-17 (PMC5437133; doi:10.1128/mSphereDirect.00187-17)
Supplement: TABLE S1 [file sph003172286st7.pdf]

Table S1: Dataset

Strain	Accession number	Isolation year	Location	RdRp genotype	Capsid genotype
Hu/GII.P16-GII.2/CUHK-NS-1082/2016/HK	KY771081	2016	HKG	GII.P16	GII.2
Hu/GII.2/CUHK-NS-1231/2016/HKG	KY421044	2016	HKG	NA	GII.2
Hu/GII.2/15-DS-4/2015/TPE	KT962983	2015	TPE	NA	GII.2
Hu/GII.P16-GII.2/Akita8/2012/JPN	LC145786	2012	JPN	GII.P16	GII.2
Hu/GII.P16-GII.2/Miyagi1/2012/JPN	LC145787	2012	JPN	GII.P16	GII.2
Hu/GII.P16-GII.2/Niigata5/2012/JPN	LC145788	2012	JPN	GII.P16	GII.2
Hu/GII.P16-GII.2/Fukui1/2012/JPN	LC145789	2012	JPN	GII.P16	GII.2
Hu/GII.P16-GII.2/Fukui2/2012/JPN	LC145790	2012	JPN	GII.P16	GII.2
Hu/GII.P16-GII.2/Saitama4/2012/JPN	LC145791	2012	JPN	GII.P16	GII.2
Hu/GII.P16-GII.2/Saitama5/2012/JPN	LC145792	2012	JPN	GII.P16	GII.2
Hu/GII.P16-GII.2/Osakacity5/2012/JPN	LC145793	2012	JPN	GII.P16	GII.2
Hu/GII.P16-GII.2/Hiroshimacity2/2012/JPN	LC145794	2012	JPN	GII.P16	GII.2
Hu/GII.P16-GII.2/Hiroshimacity1/2012/JPN	LC145795	2012	JPN	GII.P16	GII.2
Hu/GII.P16-GII.2/Ehime1/2012/JPN	LC145796	2012	JPN	GII.P16	GII.2
Hu/GII.P16-GII.2/Ehime2/2012/JPN	LC145797	2012	JPN	GII.P16	GII.2
Hu/GII.P16-GII.2/Aomori7/2014/JPN	LC145798	2014	JPN	GII.P16	GII.2
Hu/GII.P16-GII.2/Akita7/2014/JPN	LC145799	2014	JPN	GII.P16	GII.2
Hu/GII.P16-GII.2/Akita8/2014/JPN	LC145800	2014	JPN	GII.P16	GII.2
Hu/GII.P16-GII.2/Miyagi2/2014/JPN	LC145801	2014	JPN	GII.P16	GII.2
Hu/GII.P16-GII.2/Osaka4/2014/JPN	LC145802	2014	JPN	GII.P16	GII.2
Hu/GII.P16-GII.2/Osaka5/2014/JPN	LC145803	2014	JPN	GII.P16	GII.2
Hu/GII.P16-GII.2/Hiroshima6/2014/JPN	LC145804	2014	JPN	GII.P16	GII.2
Hu/GII.P16-GII.2/Hiroshimacity5/2014/JPN	LC145805	2014	JPN	GII.P16	GII.2
Hu/GII.P16-GII.2/Hiroshimacity6/2014/JPN	LC145806	2014	JPN	GII.P16	GII.2
Hu/GII.P16-GII.2/Ehime4/2014/JPN	LC145807	2014	JPN	GII.P16	GII.2
Hu/GII.P16-GII.2/Ehime5/2014/JPN	LC145808	2014	JPN	GII.P16	GII.2
Hu/GII.P22-GII.2/OsakaNI/2004/JPN	DQ366347	2004	JPN	GII.P22	GII.2
Hu/GII.P22-GII.2/OC02022/2002/JPN	AB279556	2002	JPN	GII.P22	GII.2
Hu/GII.P22-GII.2/OC01243/2001/JPN	AB279554	2001	JPN	GII.P22	GII.2
Hu/GII.Pc-GII.2/KL109/1978/MYS	JX846925	1978	MYS	GII.Pc	GII.2
Hu/GII.2/Goes28/2005/NLD	AB281089	2005	NLD	NA	GII.2
Hu/GII.P2-GII.2/OC02012/2002/JPN	AB279555	2002	JPN	GII.P2	GII.2
Hu/GII.P2-GII.2/OH08019/2008/JPN	AB662867	2008	JPN	GII.P2	GII.2
Hu/GII.P16-GII.2/OH10006/2010/JPN	AB662889	2010	JPN	GII.P16	GII.2
Hu/GII.P2-GII.2/OC08079/2008/JPN	AB662859	2008	JPN	GII.P2	GII.2
Hu/GII.2/Rotterdam39E/2002/NLD	AB281087	2002	NLD	NA	GII.2
Hu/GII.2/Heerlen7E/2002/NLD	AB281086	2002	NLD	NA	GII.2
Hu/GII.P16-GII.2/OH10025/2010/JPN	AB662899	2010	JPN	GII.P16	GII.2
Hu/GII.P16-GII.2/OH10013/2010/JPN	AB662894	2010	JPN	GII.P16	GII.2
Hu/GII.P16-GII.2/OC09072/2009/JPN	AB662870	2009	JPN	GII.P16	GII.2
Hu/GII.P2-GII.2/OH06023/2006/JPN	AB662863	2006	JPN	GII.P2	GII.2
Hu/GII.P2-GII.2/OC04038/2004/JPN	AB279557	2004	JPN	GII.P2	GII.2
Hu/GII.2/Maizuru_000602/2000/JPN	EF547398	2000	JPN	NA	GII.2
Hu/GII.P16-GII.2/OC10058/2010/JPN	AB662880	2010	JPN	GII.P16	GII.2
Hu/GII.2/Leeuwarden71/2003/NLD	AB281088	2003	NLD	NA	GII.2
Hu/GII.P2-GII.2/OC04073/2004/JPN	AB279565	2004	JPN	GII.P2	GII.2
Hu/GII.P2-GII.2/OC04071/2004/JPN	AB279564	2004	JPN	GII.P2	GII.2
Hu/GII.P2-GII.2/OC040561/2004/JPN	AB279560	2004	JPN	GII.P2	GII.2
Hu/GII.P2-GII.2/MK04/2004/JPN	DQ456824	2004	JPN	GII.P2	GII.2
Hu/GII.P16-GII.2/OH09028/2009/JPN	AB662881	2009	JPN	GII.P16	GII.2
Hu/GII.P2-GII.2/OC05143/2005/JPN	AB662852	2005	JPN	GII.P2	GII.2
Hu/GII.2/Zwolle25E/2001/NLD	AB281085	2001	NLD	NA	GII.2
Hu/GII.P2-GII.2/OC040562/2004/JPN	AB279561	2004	JPN	GII.P2	GII.2
Hu/GII.P16-GII.2/OH10026/2010/JPN	AB662900	2010	JPN	GII.P16	GII.2
Hu/GII.P16-GII.2/OH10020/2010/JPN	AB662896	2010	JPN	GII.P16	GII.2
Hu/GII.P16-GII.2/OH09032/2009/JPN	AB662884	2009	JPN	GII.P16	GII.2
Hu/GII.P16-GII.2/OC100122/2010/JPN	AB662876	2010	JPN	GII.P16	GII.2
Hu/GII.P2-GII.2/OC06005/2006/JPN	AB662854	2006	JPN	GII.P2	GII.2
Hu/GII.P2-GII.2/OC05145/2005/JPN	AB662853	2005	JPN	GII.P2	GII.2
Hu/GII.P2-GII.2/OC04043/2004/JPN	AB279559	2004	JPN	GII.P2	GII.2
Hu/GII.P2-GII.2/OC04042/2004/JPN	AB279558	2004	JPN	GII.P2	GII.2
Hu/GII.P2-GII.2/OCS030697/2004/JPN	AB279571	2004	JPN	GII.P2	GII.2

Hu/GII.P2-GII.2/OC04076/2004/JPN	AB279567	2004	JPN	GII.P2	GII.2
Hu/GII.P2-GII.2/OC04067/2004/JPN	AB279563	2004	JPN	GII.P2	GII.2
Hu/GII.P2-GII.2/OC04059/2004/JPN	AB279562	2004	JPN	GII.P2	GII.2
Hu/GII.P16-GII.2/OH100152/2010/JPN	AB662895	2010	JPN	GII.P16	GII.2
Hu/GII.2/Vaals87/2005/NLD	AB281090	2005	NLD	NA	GII.2
Hu/GII.Ph-GII.2/OC97049/1997/JPN	AB279553	1997	JPN	GII.Ph	GII.2
Hu/GII.P2-GII.2/OCS040100/2004/JPN	AB279573	2004	JPN	GII.P2	GII.2
Hu/GII.P2-GII.2/OCS040035/2004/JPN	AB279572	2004	JPN	GII.P2	GII.2
Hu/GII.P16-GII.2/OC08154/2008/JPN	AB662861	2008	JPN	GII.P16	GII.2
Hu/GII.P2-GII.2/OC04075/2004/JPN	AB279566	2004	JPN	GII.P2	GII.2
Hu/GII.2/TCH560/2002/USA	KC998960	2002	USA	NA	GII.2
Hu/GII.P16-GII.2/OH08020/2008/JPN	AB662868	2008	JPN	GII.P16	GII.2
Hu/GII.2/Leeuwarden15/2001/NLD	AB281084	2001	NLD	NA	GII.2
Hu/GII.P16-GII.2/CGMH47/2011/TWN	KC464505	2011	TWN	GII.P16	GII.2
Hu/GII.P21-GII.2/OCS020289/2002/JPN	AB279570	2002	JPN	GII.P21	GII.2
Hu/GII.P2-GII.2/Melksham/1989/GBR	X81879	1989	GBR	GII.P2	GII.2
Hu/GII.P16-GII.2/HS255/2011/USA	KJ407074	2011	USA	GII.P16	GII.2
Hu/GII.P2-GII.2/OH09030/2009/JPN	AB662883	2009	JPN	GII.P2	GII.2
Hu/GII.P2-GII.2/OH09029/2009/JPN	AB662882	2009	JPN	GII.P2	GII.2
Hu/GII.P2-GII.2/OC09094/2009/JPN	AB662871	2009	JPN	GII.P2	GII.2
Hu/GII.2/Ina/2002/JPN	AB195225	2002	JPN	NA	GII.2
Hu/GII.P2-GII.2/OC09044/2009/JPN	AB662862	2009	JPN	GII.P2	GII.2
Hu/GII.2/Coevorden191S/1999/NLD	AB281081	1999	NLD	NA	GII.2
Hu/GII.P12-GII.2/OC06014/2006/JPN	AB662856	2006	JPN	GII.P12	GII.2
Hu/GII.P12-GII.2/OC05114/2005/JPN	AB662851	2005	JPN	GII.P12	GII.2
Hu/GII.P12-GII.2/OC05041/2005/JPN	AB662850	2005	JPN	GII.P12	GII.2
Hu/GII.P21-GII.2/OC05010/2005/JPN	AB279569	2005	JPN	GII.P21	GII.2
Hu/GII.P2-GII.2/OH10029/2010/JPN	AB662901	2010	JPN	GII.P2	GII.2
Hu/GII.P2-GII.2/OH10024/2010/JPN	AB662898	2010	JPN	GII.P2	GII.2
Hu/GII.P2-GII.2/OH100112/2010/JPN	AB662892	2010	JPN	GII.P2	GII.2
Hu/GII.P2-GII.2/OH07013/2007/JPN	AB662865	2007	JPN	GII.P2	GII.2
Hu/GII.P2-GII.2/OH07001/2007/JPN	AB662864	2007	JPN	GII.P2	GII.2
Hu/GII.P2-GII.2/OH10031/2010/JPN	AB662902	2010	JPN	GII.P2	GII.2
Hu/GII.P2-GII.2/OH10007/2010/JPN	AB662890	2010	JPN	GII.P2	GII.2
Hu/GII.P2-GII.2/OH10001/2010/JPN	AB662887	2010	JPN	GII.P2	GII.2
Hu/GII.P2-GII.2/OH09035/2009/JPN	AB662886	2009	JPN	GII.P2	GII.2
Hu/GII.P2-GII.2/OC100182/2010/JPN	AB662877	2010	JPN	GII.P2	GII.2
Hu/GII.P2-GII.2/OC10009/2010/JPN	AB662875	2010	JPN	GII.P2	GII.2
Hu/GII.P2-GII.2/OH08009/2008/JPN	AB662866	2008	JPN	GII.P2	GII.2
Hu/GII.P2-GII.2/OC07107/2007/JPN	AB662858	2007	JPN	GII.P2	GII.2
Hu/GII.P16-GII.2/104320/2011/JPN	AB629946	2011	JPN	GII.P16	GII.2
Hu/GII.P12-GII.2/OC04169/2004/JPN	AB279568	2004	JPN	GII.P12	GII.2
Hu/GII.P2-GII.2/OH10008/2010/JPN	AB662891	2010	JPN	GII.P2	GII.2
Hu/GII.P2-GII.2/OH100052/2010/JPN	AB662888	2010	JPN	GII.P2	GII.2
Hu/GII.P2-GII.2/OC09104/2009/JPN	AB662873	2009	JPN	GII.P2	GII.2
Hu/GII.P2-GII.2/OH09034/2009/JPN	AB662885	2009	JPN	GII.P2	GII.2
Hu/GII.P2-GII.2/OH080292/2008/JPN	AB662869	2008	JPN	GII.P2	GII.2
Hu/GII.P2-GII.2/1014/2010/JPN	AB629941	2010	JPN	GII.P2	GII.2
Hu/GII.P2-GII.2/OC10026/2010/JPN	AB662879	2010	JPN	GII.P2	GII.2
Hu/GII.P2-GII.2/OC08124/2008/JPN	AB662860	2008	JPN	GII.P2	GII.2
Hu/GII.2/Delft48M/2000/NLD	AB281083	2000	NLD	NA	GII.2
Hu/GII.P21-GII.2/NF2002/2002/USA	JQ320072	2002	USA	GII.P21	GII.2
Hu/GII.2/DenHaag37/2000/NLD	AB281082	2000	NLD	NA	GII.2
Hu/GII.2/OC080306/2008/JPN	AB535749	2008	JPN	NA	GII.2
Hu/GII.2/Chesterfield434/1997/USA	AY054300	1997	USA	NA	GII.2
Hu/GII.Pc-GII.2/SnowMountain/1976/USA	U75682	1976	USA	GII.Pc	GII.2
Hu/GII.P16-GII.2/OH10012/2010/JPN	AB662893	2010	JPN	GII.P16	GII.2
Hu/GII.P16-GII.2/OH10021/2010/JPN	AB662897	2010	JPN	GII.P16	GII.2
Hu/GII.P12-GII.2/OC060083/2006/JPN	AB662855	2006	JPN	GII.P12	GII.2
Hu/GII.2/BUDS/2002/USA	AY660568	2002	USA	NA	GII.2
Hu/GII.Pc-GII.2/CHDC2596/1975/USA	KC597138	1975	USA	GII.Pc	GII.2
Hu/GII.P2-GII.2/OC10019/2010/JPN	AB662878	2010	JPN	GII.P2	GII.2
Hu/GII.P2-GII.2/OC09103/2009/JPN	AB662872	2009	JPN	GII.P2	GII.2
Hu/GII.P2-GII.2/OC09109-2/2009/JPN	AB662874	2009	JPN	GII.P2	GII.2
Hu/GII.P16-GII.2/16-G0904/GER	KY357453	2016	GER	GII.P16	GII.2

Hu/GII.P16-GII.2/16-G0856/GER	KY357450	2016	GER	GII.P16	GII.2
Hu/GII.P16-GII.2/16-G0907/GER	KY357451	2016	GER	GII.P16	GII.2
Hu/GII.P16-GII.2/16-G0887/GER	KY357454	2016	GER	GII.P16	GII.2
Hu/GII.P16-GII.2/16-G0901/GER	KY357462	2016	GER	GII.P16	GII.2
Hu/GII.P16-GII.2/16-G0912/GER	KY357456	2016	GER	GII.P16	GII.2
Hu/GII.P16-GII.2/16-G0880/GER	KY357452	2016	GER	GII.P16	GII.2
Hu/GII.P16-GII.2/16-G0911/GER	KY357457	2016	GER	GII.P16	GII.2
Hu/GII.P16-GII.2/16-G0905/GER	KY357455	2016	GER	GII.P16	GII.2
Hu/GII.P16-GII.2/16-G0896/GER	KY357458	2016	GER	GII.P16	GII.2
Hu/GII.P16-GII.2/16-G0831/GER	KY357449	2016	GER	GII.P16	GII.2
Hu/GII.P16-GII.2/16-G0858/GER	KY357461	2016	GER	GII.P16	GII.2
Hu/GII.P16-GII.2/16-G0895/GER	KY357460	2016	GER	GII.P16	GII.2
Hu/GII.2/16F2161/2016/CHN	KY485126	2016	CHN	NA	GII.2
Hu/GII.2/16F2149/2016/CHN	KY485125	2016	CHN	NA	GII.2
Hu/GII.2/16F2152/2016/CHN	KY485124	2016	CHN	NA	GII.2
Hu/GII.2/16SF21134/2016/CHN	KY485123	2016	CHN	NA	GII.2
Hu/GII.2/16SF21132/2016/CHN	KY485122	2016	CHN	NA	GII.2
Hu/GII.2/16SF2030/2016/CHN	KY485121	2016	CHN	NA	GII.2
Hu/GII.2/16SF2029/2016/CHN	KY485120	2016	CHN	NA	GII.2
Hu/GII.2/16SF2285/2016/CHN	KY485119	2016	CHN	NA	GII.2
Hu/GII.2/16SF2153/2016/CHN	KY485118	2016	CHN	NA	GII.2
Hu/GII.2/16SF2151/2016/CHN	KY485117	2016	CHN	NA	GII.2
Hu/GII.2/16SF2026/2016/CHN	KY485116	2016	CHN	NA	GII.2
Hu/GII.2/16SF2025/2016/CHN	KY485115	2016	CHN	NA	GII.2
Hu/GII.P2-GII.2/H7/2002/BEL	FJ409630	2002	BEL	GII.P2	GII.2
Hu/GII.P2-GII.2/OC04039/2004/JPN	AB279574	2004	JPN	GII.P2	GII.2
Hu/GII.P2-GII.2/Seoul_0460/2009/KOR	HM635168	2009	KOR	GII.P2	GII.2
Hu/GII.P2-GII.2/Seoul_0561/2009/KOR	HM635123	2009	KOR	GII.P2	GII.2
Hu/GII.P2-GII.2/Seoul1654/2011/KOR	JX439806	2011	KOR	GII.P2	GII.2
Hu/GII.P2-GII.2/Seoul1642/2011/KOR	JX439805	2011	KOR	GII.P2	GII.2
Hu/GII.P2-GII.2/Seoul1598/2011/KOR	JX439804	2011	KOR	GII.P2	GII.2
Hu/GII.P2-GII.2/Rotterdam/2009/NLD	KX446499	2009	NLD	GII.P2	GII.2
Hu/GII.P2-GII.2/SanSebastian70100349/2012/ESP	KJ504408	2012	ESP	GII.P2	GII.2
Hu/GII.P2-GII.2/IPH2161-08VG06/2008/BEL	JF697283	2008	BEL	GII.P2	GII.2
Hu/GII.P2-GII.2/SanSebastian252752/2011/ESP	KJ504407	2011	ESP	GII.P2	GII.2
Hu/GII.P2-GII.2/IPH2162-08VG06/2008/BEL	JF697284	2008	BEL	GII.P2	GII.2
Hu/GII.P2-GII.2/HMO50-201311/2013/NOR	KX019857	2013	NOR	GII.P2	GII.2
Hu/GII.P2-GII.2/NoV750/2004/CAN	EF078288	2004	CAN	GII.P2	GII.2
Hu/GII.P2-GII.2/BZ788/2012/ITA	KF475968	2012	ITA	GII.P2	GII.2
Hu/GII.P2-GII.2/Toyama_Jun44530/2010/JPN	LC147168	2010	JPN	GII.P2	GII.2
Hu/GII.P2-GII.2/Toyama_Feb-Mar23914/2010/JPN	LC147152	2010	JPN	GII.P2	GII.2
Hu/GII.P2-GII.2/Toyama_Mar23713/2010/JPN	LC147151	2010	JPN	GII.P2	GII.2
Hu/GII.P2-GII.2/Toyama_outbreakJan331/2010/JPN	LC147139	2010	JPN	GII.P2	GII.2
Hu/GII.P2-GII.2/Toyama_May42426/2010/JPN	LC147164	2010	JPN	GII.P2	GII.2
Hu/GII.P2-GII.2/Toyama_outbreakMay41825/2010/JPN	LC147163	2010	JPN	GII.P2	GII.2
Hu/GII.P16-GII.3/Smolensk_S12-31/2012/RUS	KF895841	2012	RUS	GII.P16	GII.3
Hu/GII.P16-GII.3/Novosibirsk_Nsk-N1648/2011/RUS	KF944111	2011	RUS	GII.P16	GII.3
Hu/GII.P16-GII.3/Novosibirsk_Nsk-N1659/2011/RUS	KF944110	2011	RUS	GII.P16	GII.3
Hu/GII.P16-GII.3/Omsk_O13702012/2012/RUS	KT779557	2012	RUS	GII.P16	GII.3
Hu/GII.P16-GII.13/13-BA-1/2013/TWN	KM036380	2013	TWN	GII.P16	GII.13
Hu/GII.P16-GII.4_Sydney_2012/CA3477/2015/USA	KX907727	2015	USA	GII.P16	GII.4
Hu/GII.P16-GII.4_Sydney_2012/Kawasaki194/2016/JPN	LC175468	2016	JPN	GII.P16	GII.4
Hu/GII.P16-GII.17/Saitama_T87/2002/JPN	KJ196286	2002	JPN	GII.P16	GII.17
Hu/GII.P16-GII.16/NLV_Neustrelitz260/2000/GER	AY772730	2000	GER	GII.P16	GII.16
Hu/GII.P16-GII.2/12-L/2012/TWN	KJ145320	2012	TWN	GII.P16	GII.2
Hu/GII.P16-GII.2/Wuhan_E2116/2010/CHN	JQ751039	2010	CHN	GII.P16	GII.2
Hu/GII.P16-GII.2/12-F-2/2012/TWN	KJ145321	2012	TWN	GII.P16	GII.2
Hu/GII.P16-GII.2/13/2010/CHN	KM044122	2010	CHN	GII.P16	GII.2
Hu/GII.P16-GII.2/33/2011/CHN	KM044166	2011	CHN	GII.P16	GII.2
Hu/GII.P16-GII.2/GILL70/2012/KOR	KC110856	2012	KOR	GII.P16	GII.2
Hu/GII.P16-GII.2/L53Beijing/2011/CHN	JQ889817	2011	CHN	GII.P16	GII.2
Hu/GII.P16-GII.2/Wuhan_DHGX-2/2010/CHN	JQ751041	2010	CHN	GII.P16	GII.2
Hu/GII.P16-GII.2/Seoul1921/2012/KOR	JX439906	2012	KOR	GII.P16	GII.2
Hu/GII.P16-GII.2/Wuhan_E2120/2010/CHN	JQ751040	2010	CHN	GII.P16	GII.2
Hu/GII.P16-GII.4_New_Orleans_2009/Seoul1034/2010/KOF	JX439829	2010	KOR	GII.P16	GII.4

Hu/GII.P16-GII.2/Seoul1216/2010/KOR	JX439783	2010	KOR	GII.P16	GII.2
Hu/GII.P16-GII.16/VannesL23/1999/FRA	AY682551	1999	FRA	GII.P16	GII.16
Hu/GII.P16-GII.16/Beijing55171/2008/CHN	GQ856476	2008	CHN	GII.P16	GII.16
Hu/GII.P16-GII.10/4CAU14/2012/KOR	KC110854	2012	KOR	GII.P16	GII.10
Hu/GII.P16-GII.16/Wuxi_G12/2013/CHN	KP195703	2013	CHN	GII.P16	GII.16
Hu/GII.P16-GII.13/99/2013/CHN	KM044238	2013	CHN	GII.P16	GII.13
Hu/GII.P16-GII.13/SanSebastian356037/2010/ESP	KJ156623	2010	ESP	GII.P16	GII.13
Hu/GII.P16-GII.13/Dhaka32/2011/BGD	JX683115	2011	BGD	GII.P16	GII.13
Hu/GII.P16-GII.2/09188467/2009/AUS	KP202189	2009	AUS	GII.P16	GII.2
Hu/GII.P16-GII.13/10N4439/2010/NPL	AB810005	2010	NPL	GII.P16	GII.13
Hu/GII.P16-GII.13/10N4358/2010/NPL	AB810004	2010	NPL	GII.P16	GII.13
Hu/GII.P16-GII.13/49/2011/CHN	KM044183	2011	CHN	GII.P16	GII.13
Hu/GII.P16-GII.3/SanSebastian70100075/2012/ESP	KJ504421	2012	ESP	GII.P16	GII.3
Hu/GII.P16-GII.13/Berlin1195/2012/DEU	KC832472	2012	DEU	GII.P16	GII.13
Hu/GII.P16-GII.13/09N3751/2009/NPL	AB809993	2009	NPL	GII.P16	GII.13
Hu/GII.P16-GII.16/22-2_Tokyo/1975/JPN	AB684676	1975	JPN	GII.P16	GII.16
Hu/GII.P16-GII.13/Wuxi_G7/2013/CHN	KP174834	2013	CHN	GII.P16	GII.13
Hu/GII.P16-GII.13/09N3816/2009/NPL	AB809998	2009	NPL	GII.P16	GII.13
Hu/GII.P16-GII.13/09N3798/2009/NPL	AB809997	2009	NPL	GII.P16	GII.13
Hu/GII.P16-GII.13 /HuzhouNS13045/2013/CHN	KM501037	2013	CHN	GII.P16	GII.13
Hu/GII.P16-GII.13/58/2012/CHN	KM044193	2012	CHN	GII.P16	GII.13
Hu/GII.P16-GII.13/PR1395/2012/ITA	KJ473826	2012	ITA	GII.P16	GII.13
Hu/GII.P16-GII.3/PR4526/2012/ITA	KJ473824	2012	ITA	GII.P16	GII.3
Hu/GII.P16-GII.3/SanSebastian132848/2012/ESP	KJ504423	2012	ESP	GII.P16	GII.3
Hu/GII.P16-GII.3/SanSebastian133330/2012/ESP	KJ504422	2012	ESP	GII.P16	GII.3
Hu/GII.P16-GII.13/Luckenwalde1378/2012/DEU	KC832473	2012	DEU	GII.P16	GII.13
Hu/GII.P16-GII.3/PR11068/2011/ITA	KJ473823	2011	ITA	GII.P16	GII.3
Hu/GII.P16-GII.3/SanSebastian132583/2012/ESP	KJ504419	2012	ESP	GII.P16	GII.3
Hu/GII.P16-GII.3/SanSebastian15252748/2012/ESP	KJ504418	2012	ESP	GII.P16	GII.3
Hu/GII.P16-GII.3/SanSebastian131919/2012/ESP	KJ504417	2012	ESP	GII.P16	GII.3
Hu/GII.P16-GII.13/Berlin1162/2012/DEU	KC832470	2012	DEU	GII.P16	GII.13
Hu/GII.P16-GII.2/4CAU38/2012/KOR	KC110857	2012	KOR	GII.P16	GII.2
Hu/GII.P16-GII.3/IDH2872/2010/IND	AB757784	2010	IND	GII.P16	GII.3
Hu/GII.P16-GII.13/HuzhouNS13102/2013/CHN	KM501038	2013	CHN	GII.P16	GII.13
Hu/GII.P16-GII.3/SanSebastian132719/2012/ESP	KJ504416	2012	ESP	GII.P16	GII.3
Hu/GII.P16-GII.13/Oranienburg1190/2012/DEU	KC832471	2012	DEU	GII.P16	GII.13
Hu/GII.P16-GII.16/7137_Moscow/2005/RUS	FJ383842	2005	RUS	GII.P16	GII.16
Hu/GII.P16-GII.13/IDH2770/2010/IND	AB757783	2010	IND	GII.P16	GII.13
Hu/GII.P16-GII.3/Dhaka53/2012/BGD	JX683114	2012	BGD	GII.P16	GII.3
Hu/GII.P16-GII.3/PV_Voelk0018/2013/DEU	KM289172	2013	DEU	GII.P16	GII.3
Hu/GII.P16-GII.4/20160118_EL03LP2/2016/KOR	KX764848	2016	KOR	GII.P16	GII.4
Hu/GII.P16-GII.4/20160118_EL03ALP2/2016/KOR	KX764849	2016	KOR	GII.P16	GII.4
Hu/GII.P16-GII.4/20160118_EL03BLP2/2016/KOR	KX764850	2016	KOR	GII.P16	GII.4
Hu/GII.P16-GII.4/20160118_EL06LP1/2016/KOR	KX764851	2016	KOR	GII.P16	GII.4
Hu/GII.P16-GII.4/20160122_FL01ALP1/2016/KOR	KX764852	2016	KOR	GII.P16	GII.4
Hu/GII.P16-GII.4/20160122_FL01CLP1/2016/KOR	KX764853	2016	KOR	GII.P16	GII.4
Hu/GII.P16-GII.4/20160122_FL01DLP1/2016/KOR	KX764854	2016	KOR	GII.P16	GII.4
Hu/GII.P16-GII.4/20160122_FL07LP2/2016/KOR	KX764855	2016	KOR	GII.P16	GII.4
Hu/GII.P16-GII.4/20160122_FL07ALP3/2016/KOR	KX764856	2016	KOR	GII.P16	GII.4
Hu/GII.P16-GII.2/16F2284/2016/CHN	KY485110	2016	CHN	GII.P16	GII.2
Hu/GII.P16-GII.2/16F21134/2016/CHN	KY485113	2016	CHN	GII.P16	GII.2
Hu/GII.P16-GII.2/16F2029/2016/CHN	KY485112	2016	CHN	GII.P16	GII.2
Hu/GII.P16-GII.2/16F2026/2016/CHN	KY485111	2016	CHN	GII.P16	GII.2
Hu/GII.P16-GII.2/16F2025/2016/CHN	KY485109	2016	CHN	GII.P16	GII.2
Hu/GII.P16-GII.2/16F21132/2016/CHN	KY485108	2016	CHN	GII.P16	GII.2
Hu/GII.P16-GII.2/16F2285/2016/CHN	KY485114	2016	CHN	GII.P16	GII.2
Hu/GII.P16-GII.2/16F2019/2016/CHN	KY485107	2016	CHN	GII.P16	GII.2

NA: Not avaiable
