# Supplementary material for: Phylogenetic Analyses Suggest that Factors Other Than the Capsid Protein Play a Role in the Epidemic Potential of GII.2 Norovirus
Source: mSphere. 2017 May 17;2(3):e00187-17. doi: 10.1128/mSphereDirect.00187-17 (PMC5437133; doi:10.1128/mSphereDirect.00187-17)
Supplement: TABLE S2 [file sph003172286st8.pdf]

Table S2. Codon position of complete VP1 sequences (1629 nt) with positive selection

Positively seleted site	Method (p value)	Domain
24	MEME[1] (p=0.025)	N-terminal
78	iFEL[2] (p=0.018), MEME (p=0.044)	Shell
99	MEME (p=0.021)	Shell
275	MEME (p=0.012)	P1
344	MEME (p=0.036)	P2 (surface)
345	iFEL (p<0.01), MEME (p=0.019)	P2 (surface)
354	MEME (p=0.047)	P2 (surface)
384	MEME (p<0.01)	P2 (surface)
385	MEME (p=0.023)	P2 (surface)

1. Mixed Effect Model of Evolution, 2. internal Fixed Effect Likelihood methods
