# Supplementary material for: Phylogenetic Analyses Suggest that Factors Other Than the Capsid Protein Play a Role in the Epidemic Potential of GII.2 Norovirus
Source: mSphere. 2017 May 17;2(3):e00187-17. doi: 10.1128/mSphereDirect.00187-17 (PMC5437133; doi:10.1128/mSphereDirect.00187-17)
Supplement: TABLE S3 [file sph003172286st9.pdf]

Table S3: Amino acid substitution observed in the RdRp sequence of reemerging GII.P16 strains

	293	332	357	360
Reemerging GII.P16 (2016)	T	I	Q	A
P16 cluster[1] (2008-2014)	S	V	K	T
P2-P16 mix cluster[1] (2009-2013) (without reemerging strains)	S	V	K	T

1. Phylogenetic clustering shown in Fig. 4, assigned based on the phylogenetic clustering of VP1 encoding sequence and the associated polymerase genotype
