# Supplementary material for: Phylogenetic Analyses Suggest that Factors Other Than the Capsid Protein Play a Role in the Epidemic Potential of GII.2 Norovirus
Source: mSphere. 2017 May 17;2(3):e00187-17. doi: 10.1128/mSphereDirect.00187-17 (PMC5437133; doi:10.1128/mSphereDirect.00187-17)
Supplement: TABLE S4 [file sph003172286st10.pdf]

Table S4: Evolutionary models used in BEAST analyses

Sequence	Genotype	Dataset	#. Sequences	Length (nt)	Substitution model	Clock model	Tree prior
VP1, partial	GII.2	All	151	1337	TN93+G+I	Strict	Bayesian Skyline
		P2 cluster	47	1337	TN93+G	Strict	Bayesian Skyline
		P2-P16 mix cluster	36	1337	TN93+G+I	Strict	Bayesian Skyline
		P16 cluster	38	1337	TN93+G+I	Strict	Bayesian Skyline
VP1, complete	GII.2	All	134	1629	TN93+G+I	Strict	Bayesian Skyline
		P2 cluster	47	1629	TN93+G	Strict	Bayesian Skyline
		P2-P16 mix cluster	23	1629	TN93+G	Strict	Exponential
		P16 cluster	36	1629	TN93+G	Strict	Bayesian Skyline
RdRp, partial	GII.P2	All	72	720	K2P+G	Strict	Bayesian Skyline
	GII.P16	All	131	720	K2P+G	Strict	Bayesian Skyline
		Without reemerging GII.P16	99	720	K2P+G	Strict	Exponential
